# Antiproliferative and pro-apoptotic effects of *Pseudevernia furfuracea* (L.) Zopf extract and its active component physodic acid via oxidative stress and DNA damage in breast cancer cells

**DOI:** 10.3389/fonc.2025.1557884

**Published:** 2025-05-21

**Authors:** Dominika Sebova, Simona Zilakova, Viktoria Medvecova, Michal Goga, Richard Frenak, Annamaria Bardelcikova, Andrej Mirossay, Ladislav Mirossay, Jan Mojzis, Martin Kello

**Affiliations:** ^1^ Department of Pharmacology, Faculty of Medicine, Pavol Jozef Šafárik University, Kosice, Slovakia; ^2^ Department of Botany, Institute of Biology and Ecology, Faculty of Science, Pavol Jozef Šafárik University, Kosice, Slovakia; ^3^ Center for Interdisciplinary Biosciences, Technology and Innovation Park, Pavol Jozef Šafárik University, Kosice, Slovakia

**Keywords:** *Pseudevernia furfuracea*, physodic acid, breast cancer, apoptosis, oxidative damage, immune checkpoints

## Abstract

**Background:**

Mammary gland malignancies are the most diagnosed oncological diseases in women. The currently available treatment faces several problems, including resistance to cytostatics and the relatively high recurrence rates. These limitations have led to an increasing interest in natural substances as potential anticancer agents. Therapeutic approaches using a combination of natural anticancer agent and conventional cytostatic drug could also be beneficial in minimising the risk of chemotherapy. In the present study, we evaluated the anticancer effect of *Pseudevernia furfuracea* (L.) Zopf extract (PSE) and isolated the secondary metabolite physodic acid (PHY) in *in vitro* models of breast cancer subtypes (ER+, HER2+, and triple negative).

**Methods:**

To investigate the effects of tested compounds, a range of assays were employed. BrdU and clonogenic assays were used to evaluate antiproliferative activity. Flow cytometry and Western blot were used to demonstrate apoptotic cell death, oxidative stress, DNA damage, and immune checkpoint modulation in a time-dependent manner (24, 48, and 72 h).

**Results:**

PSE and PHY induced cycle arrest at a G1 checkpoint with modulation of cell cycle-related proteins. Furthermore, activation of intrinsic apoptotic pathway, involving changes in Bcl-2 family proteins, caspase-3/-7 activity, caspase-9 cleavage, cytochrome *c* release, and PARP cleavage, was detected in all BC cells. Moreover, we determined the PSE- and PHY-mediated generation of ROS and RNS, which led to DNA damage and the activation of the DNA damage response.

**Conclusions:**

Treatment with PSE and PHY in BC cells resulted in mitochondrial apoptosis associated with oxidative stress and DNA damage. Furthermore, modulation of immune checkpoint PD-1/PD-L1 was demonstrated. Based on the results, we assume the use of PSE and PHY as promising targeted agents for BC.

## Highlights

- PSE and PHY reduced the viability of the tested breast cancer cells (ER+, HER2+, and triple negative).
- PSE and PHY induced apoptosis.
- PSE and PHY activated the mitochondrial apoptotic pathway.
- PSE and PHY induced oxidative stress and DNA damage.
- PSE and PHY modulated the expression of PD-L1.

## 1 Introduction

Breast cancer (BC) represents one of the most frequently diagnosed cancer type in women worldwide. Despite advances in diagnostic and therapeutic approaches, the IARC (International Agency for Research of Cancer) statistic data indicates a global increase in newly diagnosed cancer cases. According to the latest data from the GLOBOCAN database in 2022, the incidence of BC was estimated at approximately 2.3 million cases, while mortality rates showed about 666,000 deaths associated with BC (1).

The effectiveness of current BC treatments is often compromised by tumour heterogeneity, resistance to treatment, and the high recurrence rates, particularly in aggressive subtypes such as HER2-positive and triple-negative breast cancer (TNBC). These challenges underscore the urgent need for novel, targeted anticancer agents that can overcome these limitations and improve patient outcomes (2).

Natural products have long been a rich source of bioactive compounds with potential anticancer properties capable of modulating various biological targets (3). Many studies on biomolecules suggest their potential effectiveness against tumour cells (4). Species of *Taxus*, *Vinca*, *Camptotheca*, or *Podophyllum* are a few examples of natural sources of anticancer drugs, which are already used as clinical therapeutics.

Nowadays, lichens represent a relatively unexplored reservoir of unique secondary metabolites with potential therapeutic applications. This symbiotic association of mycobiont and photobiont and/or cyanobiont is characterised by the production of secondary metabolites, with diverse chemical structure (depsides, depsidones, phenolic compounds, diterpenes, etc.) exhibiting various pharmacological effects, including anti-inflammatory, antiviral, antibacterial, antimycotic, antipyretic, analgesic, and anticancer activity (5–7). Moreover, we and others have documented the cytotoxic and antiproliferative activities of various lichens [e.g., *Pseudevernia furfuracea* (L.) Zopf, *Evernia prunastri* (L.) Ach., *Stereocaulon tomentosum* (L.) Fr., etc.] and their secondary metabolites (e.g., physodic acid, evernic acid, atranorin, gyrophoric acid, etc.) in a broad spectrum of *in vitro* cancer models (cervix, lung, colon, ovarian, breast cancer, etc.) (5, 6, 8, 9). Our previous work that focused on the *Pseudevernia furfuracea* effect on leukaemia cells showed induction of apoptosis, ROS production, DNA damage, modification of DNA repair mechanisms, and modulation of MAPK signalling (5). Focusing on BC, our results demonstrate that both, *Pseudevernia furfuracea* extract (PSE) and isolated secondary metabolite, physodic acid (PHY), inhibit the metabolism of BC cell lines (MCF-7, MDA-MB-231) in a concentration-dependent manner (8). Furthermore, Petrova et al. (2021) studied the possible modulation of the tumour microenvironment by PSE and PHY in MCF-10A, BJ-5ta, and HUVECs (endothelial cells) cell lines. Published data show an influence of both samples on the expression of proteins associated with epithelial–mesenchymal transition (N-cadherin, fibronectin, α-SMA, Slug, Smad 2/3), while PSE showed higher efficacy compared to PHY alone, probably due to the synergism of several components contained in the acetone extract. Furthermore, inhibition of angiogenesis was demonstrated (10).

However, further studies of molecular mechanisms are needed for many lichen species, including *Pseudevernia furfuracea*, which remains relatively understudied in the context of cancer research. Therefore, this study aims to evaluate the mechanism underlying the proapoptotic and antiproliferative effects of PSE and PHY in an *in vitro* model focused on three molecular subtypes of BC. Antiproliferative properties, induction of apoptosis (activation of mitochondrial pathway, cell cycle distribution, mitochondrial dysfunction), oxidative stress, DNA damage, and PD-L1 changes mediated by PSE and PHY are the main subjects of this study.

## 2 Materials and methods

### 2.1 Lichen material, extraction, and isolation

The collection and identification of lichen *Pseudevernia furfuracea* (L.) Zopf was conducted by Assoc. Prof. Goga (Department of Botany, Institute of Biology and Ecology, Faculty of Science, Pavol Jozef Šafárik University). *Pseudevernia furfuracea* (L.) Zopf extract was prepared by acetone extraction and characterised by HPLC and NMR spectroscopy as published in our previous research paper (10). The secondary metabolite, physodic acid, was isolated from that sample (11). Both PSE and PHY were diluted in DMSO (Sigma Aldrich, St. Louis, MO, USA) to prepare stock solutions (final dilutant v/v % in cell culture medium <0.02%).

### 2.2 Cell lines and culture conditions

As *in vitro* BC models, MCF-7 (HTB-22™, human breast adenocarcinoma), MDA-MB-231 (HTB-26™, human triple-negative breast carcinoma) and SK-BR-3 (HTB-30™, human HER2+ breast carcinoma) cell lines (ATCC, Manassas, VA, USA) were used. Cells were cultured in high-glucose Dulbecco's modified Eagle's medium (DMEM; Biosera, Kansas City, MO, USA) supplemented with 10% foetal bovine serum (FBS; Biosera, Kansas City, MO, USA) and 1% antibiotic/antimycotic solution (Merck, Darmstadt, Germany). Non-cancer cell models were represented by MCF-10A (CRL-10317™, human mammary gland epithelial cells) and BJ-5ta (CRL-4001™, human dermal fibroblasts) cell lines. MCF-10A cells were cultured in high-glucose DMEM F12 Medium supplemented with insulin, EGF, HC (epithelial growth factor, hydrocortisone; Merck, Darmstadt, Germany) and 10% FBS (Gibco, Thermo Scientific, Rockford, IL, USA), while the BJ-5ta cell line was maintained in a medium mixture composed of high-glucose DMEM:M199 4:1 (Biosera, Kansas City, MO, USA), Hygromycin B (0.01 mg/ml; Merck, Darmstadt, Germany), and 10% FBS (Gibco, Thermo Scientific, Rockford, IL, USA). Cells were cultured at 37°C in a humidified incubator under 5% CO_2_ atmosphere (8).

### 2.3 BrdU proliferation assay

The antiproliferative effect of the tested compounds was assessed by BrdU colorimetric assay (BrdU Colorimetric kit, Roche Diagnostics GmbH, Mannheim, Germany). Cells were seeded and cultured in 96-well culture plates at a density of 5 × 10^3^ per well at 37°C, 5% CO_2_ for 24 h. After 24 h, cells were treated with PSE and PHY, respectively, and DMSO vehicle in a concentration range of 25–100 µmol/L. The cells were incubated for another 48 h and then labelled with BrdU labelling solution (1:300) and incubated at 37°C in 5% CO_2_ atmosphere overnight. The cells were fixed by adding a fixing/denaturing solution, washed, and incubated with BrdU detection antibody at room temperature for 1 h. After 1 h, cells were washed, and anti-mouse HRP-linked antibody solution (1:2,000) was added, followed by 1 h of incubation at room temperature. Then, cells were washed by adding wash buffer (repeated three times) and incubated with TMB substrate for 5 min. A change to a blue colour was observed, and the absorbance was measured at a wavelength of 650 nm using the automated Cytation™ 3 Cell Imaging Multi-Mode Reader (Biotek, Winooski, VT, USA). The reaction was stopped by adding 1 M H_2_SO_4_ solution to each well, while colour changes to yellow was observed. The change in absorbance was measured at a wavelength of 450 nm. Based on 50% inhibition of cell growth compared to the control, IC_50_ values were determined. For the calculation of the IC_50_ values, the relative predictive TREND function was used (10).

### 2.4 Clonogenic assay

BC cell lines were seeded and cultured in six-well plates at a density of 1 × 10^5^ cells per well for 24 h. After 24 h, cells were treated with PSE, PHY, and DMSO vehicle (IC_50_ concentrations) and incubated at 37°C, 5% CO_2_ for 24, 48, and 72 h. After exposure to tested compounds at the indicated time endpoints, cells were trypsinised, and the cell density was adjusted to 1 × 10^4^ cells per well, followed by 10 days of incubation under standard conditions. After 10 days, colonies were fixed (4% paraformaldehyde, Merck, Darmstadt, Germany) and stained with 0.08% methylene blue (Merck, Darmstadt, Germany) in 70% methanol solution for 15 min. Then, cells were washed with PBS and dried. Scans were obtained using the HP Scanjet G3010 Photo Scanner (Hewlett-Packard, Palo Alto, CA, USA). For colony counting, an iBright™ FL1500 Imaging System (Thermo Scientific, Rockford, IL, USA) and iBright Analysis software (version 5.2.2, Thermo Fisher Scientific, Cleveland, OH, USA, RRID : SCR_017632) were used (12).

### 2.5 Western blot

To evaluate changes in protein levels, WB analyses were performed. Cells were seeded in large Petri dishes at a density of 1 × 10^6^ cells per dish and incubated with PSE, PHY, and DMSO vehicle for 24, 48, and 72 h. Cell lysates were prepared using Laemmli lysis buffer [glycerol, 20% sodium dodecyl sulphate (SDS), 1 M Tris/HCl (pH = 8.6), deionised H_2_O, protease, and phosphatase inhibitors] (all components from Merck, Darmstadt, Germany). Further cell disruption was obtained by sonication. Protein concentrations were determined for each sample using a colorimetric Pierce^®^ BCA protein assay kit (Thermo Scientific, Rockford, IL, USA). The absorbance was measured at a wavelength of 570 nm using the automated Cytation™ 3 Cell Imaging Multi-Mode Reader (Biotek, Winooski, VT, USA). The samples were loaded onto 10% SDS-PAA gel (25 ng of protein per well), and protein separation by electrophoresis was performed. Then, proteins were transferred to a PVDF (polyvinylidene difluoride) membrane (Thermo Scientific, Rockford, IL, USA) using a dry transfer system (iBlot™ dry blotting system, Thermo Scientific, Rockford, IL, USA). To minimise nonspecific binding, membranes were blocked in 5% bovine serum albumin solution (BSA; SERVA, Heidelberg, Germany) or 5% dry nonfat milk solution (Cell Signaling Technology^®^, Danvers, MA, USA) for 1 h at room temperature. Five percent BSA and 5% milk solutions were prepared with TBS-Tween (pH = 7.4). After 1 h, the membranes were washed with TBS-Tween and incubated with primary antibody (**Supplementary Table S1**) overnight at 4°C, followed by washing with TBS-Tween (3× 5 min), and incubated with horseradish peroxidase-conjugated secondary antibody (**Supplementary Table S1**) for 1 h at room temperature. Protein expression was detected using ECL chemiluminescent substrate (Thermo Scientific, Rockford, IL, USA) and iBright™ FL1500 Imaging System (Thermo Scientific, Rockford, IL, USA). Protein expression was normalised to total protein levels using No-Stain™ Protein Labelling Reagent (Thermo Scientific, Rockford, IL, USA). The iBright Analysis software (version 5.2.2, Thermo Fisher Scientific, Cleveland, OH, USA, RRID : SCR_017632) was used for normalisation and quantification of protein expression (10).

### 2.6 JC-1 mitochondrial membrane potential assay

JC-1 dye occurs at low concentrations in a monomeric form exhibiting a green fluorescence signal. With MMP-dependent accumulation in active mitochondria, JC-1 forms aggregates emitting red fluorescence. Therefore, mitochondrial depolarisation is indicated by changes in the ratio of red-to-green fluorescence. BC cells were seeded and cultured in 96-well culture plates at a density of 5 × 10^3^ cells per well under standard conditions. After 24 h, cells were treated with PSE and PHY, respectively, and DMSO vehicle and incubated at 37°C in 5% CO_2_ atmosphere for 24, 48, and 72 h. After incubation time, cells were fixed with 4% paraformaldehyde (Merck, Darmstadt, Germany) for 15 min and permeabilised with 0.5% Tween solution (Merck, Darmstadt, Germany) for 10 min. The cells were washed with PBS and incubated with the JC-1 working solution (final concentration 2.5 µg/ml, Enzo Life Sciences) for 30 min in the dark at room temperature. Cells were washed with PBS twice, and fluorescence was visualised at wavelengths of 590 nm/530 nm using the automated Cytation™ 3 Cell Imaging Multi-Mode Reader (Biotek, Winooski, VT, USA) (13).

### 2.7 Flow cytometry

For flow cytometric analyses, BC cell lines were seeded in middle Petri dishes at a density of 25 × 10^4^ cells per dish and cultured for 24 h under standard conditions. Then, cells were treated with tested compounds, followed by incubation time according to analysis. After each exposure time, cells were harvested, centrifuged, and pellets were obtained. Pellets were resuspended in PBS and divided for multiple analyses. For fluorescence measurement, the FACSCalibur flow cytometer (Becton Dickinson, San Jose, CA, USA) was used. Flow cytometry analyses data were evaluated using Flow Jo 10.0 software (Tree Star, Inc., Ashland, OR, USA, RRID : SCR_008520). In software analyses, the measured cell population density plot was cleaned of debris and disintegrated cells through the SSC (side scatter, granularity) vs. FSC (forward scatter, size) properties. Gating was then performed on the basis of fluorescence detection in FL channels to distinguish positive or negative populations in particular analyses. The percentage of cells with positive autofluorescence (AF) signal was set as background, and the final positive population was calculated after AF subtraction (5).

#### 2.7.1 Cell cycle analysis

The distribution of cells in the cell cycle phases was assessed by measuring the amount of propidium iodide-stained DNA in treated cells. After each exposure time, cells were harvested, centrifuged, resuspended in PBS, and fixed with cold 70% ethanol. The samples were stored at −20°C until analysis. On the day of analysis, samples were centrifuged, incubated with staining solution (0.5 mg/ml of ribonuclease A, a 0.2% final concentration of Triton X-100, and 0.025 mg/ml of PI in 500 µl of PBS (all from Sigma Aldrich, St. Louis, MO, USA) for 30 min at room temperature in the dark and analysed. Cell cycle distribution was determined by deconvolution of DNA content histograms, after discrimination of doublets (FL-2-W vs FL-2-A) and other cellular aggregates, using the Dean–Jett–Fox model (5).

#### 2.7.2 Detection of p21/WAF1 expression

P21/Waf1 expression was determined using p21 Waf1/Cip1 (12D1) Rabbit mAb (1:200, Cell Signaling Technology, Danvers, MA, USA). Prior to analysis, samples were fixed with 4% paraformaldehyde (Merck, Darmstadt, Germany) for 15 min, washed with PBS, and permeabilised using 0.5% Tween solution (Merck, Darmstadt, Germany) for 10 min. Then, cells were incubated with secondary antibody conjugate for 30 min at room temperature in the dark and analysed. The positive vs. negative populations were gated in the FL-2 (585/42) channel after SSC vs. FSC exclusions of debris and death cells (5).

#### 2.7.3 Detection of mitochondrial membrane potential changes

For detection of MMP changes, accumulation of positively charged tetramethyl rhodamine ethyl ester (TMRE; 1:100, Sigma-Aldrich, St. Louis, MO, USA) probe in active mitochondria was used. The obtained pellets were resuspended in PBS, cells were incubated with TMRE for 30 min at room temperature in the dark, and flow cytometry analysis was performed. The positive vs. negative population was gated in the FL-2 (585/42) channel after SSC vs. FSC exclusion of debris and death cells and visualised as a single parameter density plot. The percentage of cells with dissipated MMP was calculated (5).

#### 2.7.4 Annexin V/PI staining

Annexin V is a fluorescent dye that specifically binds to a phosphatidylserine, an anionic phospholipid, localised on the cell surface during the early stage of apoptosis, while propidium iodide (PI) intercalates DNA only in cells with membrane integrity loss. Therefore, Annexin V/PI double staining is often used for the separation of living cells (An−PI−), cells in an early stage of apoptosis (An+PI−), cells in a late stage of apoptosis (An+PI+), and dead cells (An−PI+) in a cell population. Cells were treated, harvested, centrifuged, and pellets were resuspended in PBS. The samples were incubated with Annexin V-Alexa Fluor^®^ 647 (1:100, Thermo Scientific, Rockford, IL, USA) for 30 min at room temperature in the dark. Then, propidium iodide (5 mg/ml, 1:500, Sigma-Aldrich, Steinheim, Germany) was added, and samples were analysed. The measured cell population was analysed through the FL-2 (585/42) vs. FL-4 (661/16) channels and visualised as a two-parameter density plot. The percentage of cells in all quadrants was calculated (5).

#### 2.7.5 Analysis of cytochrome *c* release

Cytochrome *c* release was analysed using cytochrome *c* antibody (6H2) FITC (Invitrogen, Carlsbad, CA, USA). Prior to analysis, samples were fixed with 4% paraformaldehyde (Merck, Darmstadt, Germany) for 15 min, washed with PBS, and permeabilised using 0.5% Tween solution (Merck, Darmstadt, Germany) for 10 min. Then, cells were incubated with cytochrome *c* antibody (6H2) FITC (1:100) for 30 min at room temperature in the dark. The samples were centrifuged, and obtained pellets were resuspended in PBS and analysed. The positive vs. negative populations were gated in the FL-1 (530/30) channel after SSC vs. FSC exclusions of debris and death cells (14).

#### 2.7.6 Detection of caspase-3/-7 activity

Caspase-3/-7 activity was evaluated using CellEvent™ Caspase-3/7 Green Detection Reagent (Thermo Scientific, Rockford, IL, USA), which was added to samples, followed by incubation for 30 min at 37°C in the dark. During apoptosis, the caspase 3 and 7 proteins are activated and able to cleave the caspase-3/-7 recognition sequence encoded in the DEVD peptide (conjugated to a nucleic acid-binding dye). Cleavage of the recognition sequence and binding of DNA by the reagent labels the apoptotic cells with a bright, fluorogenic signal that has absorption/emission maxima of ∼511/533 nm (FL-1). After 30 min, cells were stained using SYTOX™ AADvanced™ Dead Cell Stain (final concentration 1 µM, Thermo Scientific, Rockford, IL, USA), and samples were consequently analysed. The measured cell population was analysed through the FL-1 (530/30) vs. FL-4 (661/16) channels and visualised as a two-parameter density plot. The percentage of cells in all quadrants was calculated (5).

#### 2.7.7 Detection of caspase-9 activation

Caspase-9 cleavage was determined using Cleaved Caspase-9 (Asp315) (D8I9E) Rabbit mAb PE (1:200, Cell Signaling Technology, Danvers, MA, USA). Prior to analysis, samples were fixed with 4% paraformaldehyde (Merck, Darmstadt, Germany) for 15 min, washed with PBS, and permeabilised using 0.5% Tween solution (Merck, Darmstadt, Germany) for 10 min. After permeabilisation, cells were incubated with antibody conjugate for 30 min at room temperature in the dark and analysed. The positive vs. negative populations were gated in the FL-2 (585/42) channel after SSC vs. FSC exclusions of debris and death cells (14).

#### 2.7.8 Analysis of Bcl-2 family protein expression

Changes in Bcl-2 family protein expression were determined using Anti-Bad antibody (Y208, Dylight^®^488, (1:200, Abcam), phospho-Bad (Ser112, 40A9) Rabbit monoclonal antibody PE (1:200, Cell Signaling Technology, Danvers, MA, USA), and Bcl-xL (54H6) Rabbit monoclonal antibody Alexa 488 (1:200, Cell Signaling Technology, Danvers, MA, USA). Prior to analysis, samples were fixed with 4% paraformaldehyde (Merck, Darmstadt, Germany) for 15 min, washed with PBS, and permeabilised using 0.5% Tween solution (Merck, Darmstadt, Germany) for 10 min. Then, cells were incubated with primary antibody conjugate for 30 min at room temperature in the dark and analysed. The positive vs. negative populations were gated in the FL-1 (530/30) or FL-2 (585/42) channel after SSC vs. FSC exclusions of debris and death cells (14, 15).

#### 2.7.9 Detection of DNA damage markers

Prior to analyses, samples were fixed with 4% paraformaldehyde (Merck, Darmstadt, Germany) for 15 min, washed with PBS, and permeabilised using 0.5% Tween solution (Merck, Darmstadt, Germany) for 10 min. Cells were incubated with primary antibodies [Anti-Oxoguanine 8 antibody, 1:50, Abcam, Phospho-Histone H2A.X (Ser139, 20E3) Rabbit mAb Alexa Fluor^®^ 647, 1:50, Cell Signaling Technology, Danvers, MA, USA] for 30 min at room temperature in the dark, followed by centrifugation and incubation with secondary antibody (1:300, Alexa fluor 633, Thermo Scientific, Rockford, IL, USA) for 15 min (use of secondary antibody refers only to 8-oxoguanine analysis). The samples were centrifuged, and pellets were resuspended in PBS and analysed. The positive vs. negative populations were gated in the FL-4 (661/16) channel after SSC vs. FSC exclusions of debris and death cells (16).

#### 2.7.10 Detection of ROS and RNS

To evaluate changes in ROS and RNS production, cells were treated, harvested, and centrifuged. The obtained pellets were resuspended in PBS, followed by incubation with MitoSOX™ Red mitochondrial superoxide indicator (final concentration 5 µM, Thermo Scientific, Rockford, IL, USA) for detection of intracellular production of oxygen radicals for 15 min. To evaluate the production of reactive nitrogen species, cells were incubated with DAF-FM DA (4-amino-5-methylamino-2′,7′-difluorofluorescein diacetate) (final concentration 2 mM, Merck, Darmstadt, Germany). After 15 min of incubation, flow cytometry analyses were performed. The positive vs. negative populations were gated in the FL-1 (530/30) or FL-2 (585/42) channel after SSC vs. FSC exclusions of debris and death cells (5, 10, 16).

### 2.8 Statistical analyses

Results are expressed as mean ± SD. Statistical analyses were performed using one-way ANOVA with Dunnett's *post hoc* test. All experimental data were analysed using GraphPad Prism (version 9.0.2., La Jolla, California, USA; RRID : SCR_002798). p-Values <0.05 (vs. DMSO vehicle) were considered statistically significant. All experiments were performed in independent replicates.

## 3 Results

### 3.1 Antiproliferative activity of PSE and PHY

**Figure 1:**
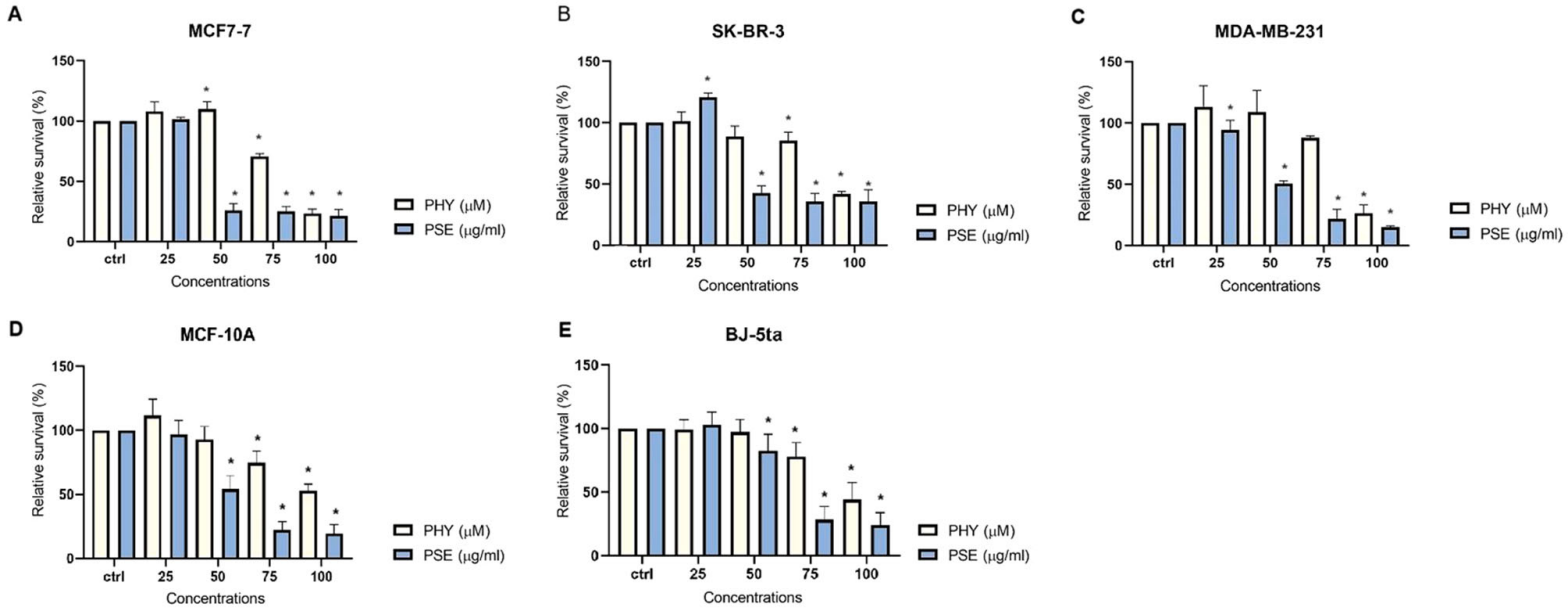
PHY- and PSE-mediated inhibition of cell proliferation. MCF-7 **(A)**, SK-BR-3 **(B)**, MDA-MB-231 **(C)**, MCF-10A **(D)**, and BJ-5ta **(E)** cells were treated with PHY (25–100 µM) and PSE (25–100 µg/ml) for 72 (h) Relative survival values are expressed as mean ± standard deviation of independent experiments (*p < 0.05 compared to control, based on ordinary one-way ANOVA with Dunnett's *post hoc* test).

The antiproliferative effects of PSE and PHY were studied using BrdU assay, flow cytometry, and clonogenic assay. The results revealed a suppression of cell proliferation activity in PSE and PHY-treated cell lines in a time- and a dose-dependent manner. PSE exhibited greater antiproliferative activity compared to PHY, likely due to the synergistic effects of its constituent components (**Figure 1**). The most potent *Pseudevernia furfuracea*-mediated inhibition of proliferation was observed in the MCF-7 cell line (IC_50_: 85.35 ± 0.24 µM/42.05 ± 1.2 µg/ml), followed by MDA-MB-213 (IC_50_: 89.56 ± 3.06 µM/53.82 ± 1.13 µg/ml), and SK-BR-3 (IC_50_: 93.77 ± 3.02 µM/47.62 ± 3.57 µg/ml). In the MCF-10A and BJ-5ta cell lines, which serve as non-cancer *in vitro* models, the IC_50_ values ranged from 110.67 ± 6.65 to 112.01 ± 8.75 µM for PHY and from 66.3 ± 0.67 to 72.2 ± 2.98 µg/ml for PSE (**Table 1**). The DMSO vehicle did not show any inhibition of cell proliferation in the tested concentration range, with relative survival rates ≥100% for each cell line.

**Table 1:** Calculated IC_50_ for PHY and PSE against BC cell lines.

IC_50_	MCF-7	MDA-MB-231	SK-BR-3	MCF-10A	BJ-5ta
PHY (µM)	85.35 ± 0.24	89.56 ± 3.06	93.77 ± 3.02	110.67 ± 6.65	112.01 ± 8.75
PSE (µg/ml)	42.05 ± 1.2	53.82 ± 1.13	47.62 ± 3.57	66.3 ± 0.67	72.2 ± 2.98

Results are presented as mean ± SD of independent experiments.

**Figure 2:**
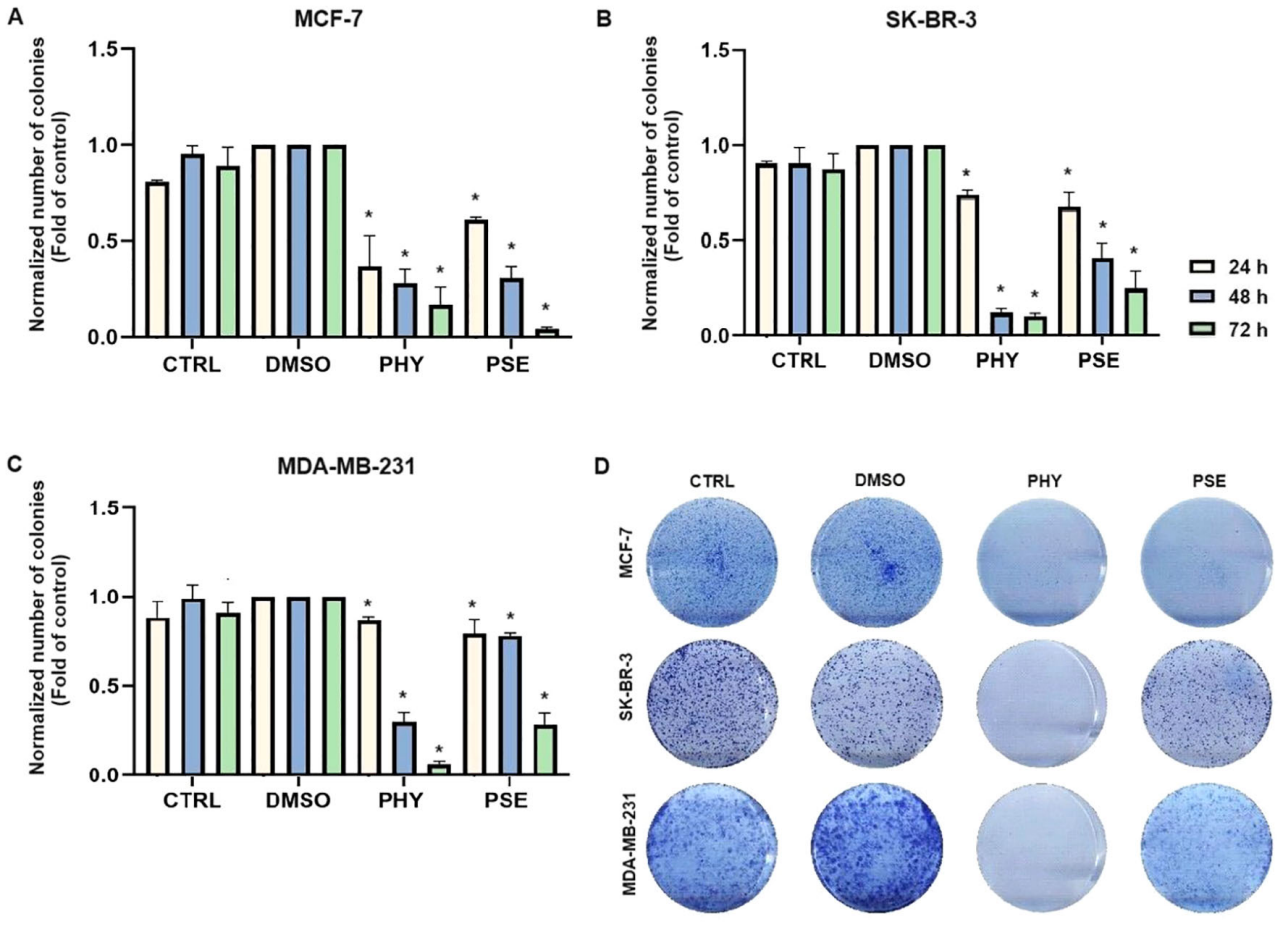
PHY- and PSE-mediated suppression of clonogenicity in MCF-7 **(A)**, SKBR-3 **(B)**, and MDA-MB-231 **(C)** cell lines. Cells were treated with IC_50_ values of PHY and PSE for 24, 48, and 72 (h) Representative images of clonogenic potential after 48 h of treatment of cells are presented in **(D)**. Results are expressed as mean ± standard deviation of independent experiments (*p < 0.05 compared to DMSO control, based on ordinary one-way ANOVA with Dunnett's *post hoc* test).

The results obtained from clonogenic assay are presented in **Figure 2** and clearly show a suppression of colony formation ability in MCF-7, SK-BR-3, and MDA-MB-231 cells treated with PSE and PHY.

### 3.2 Cell cycle distribution after PSE/PHY treatment

**Figure 3:**
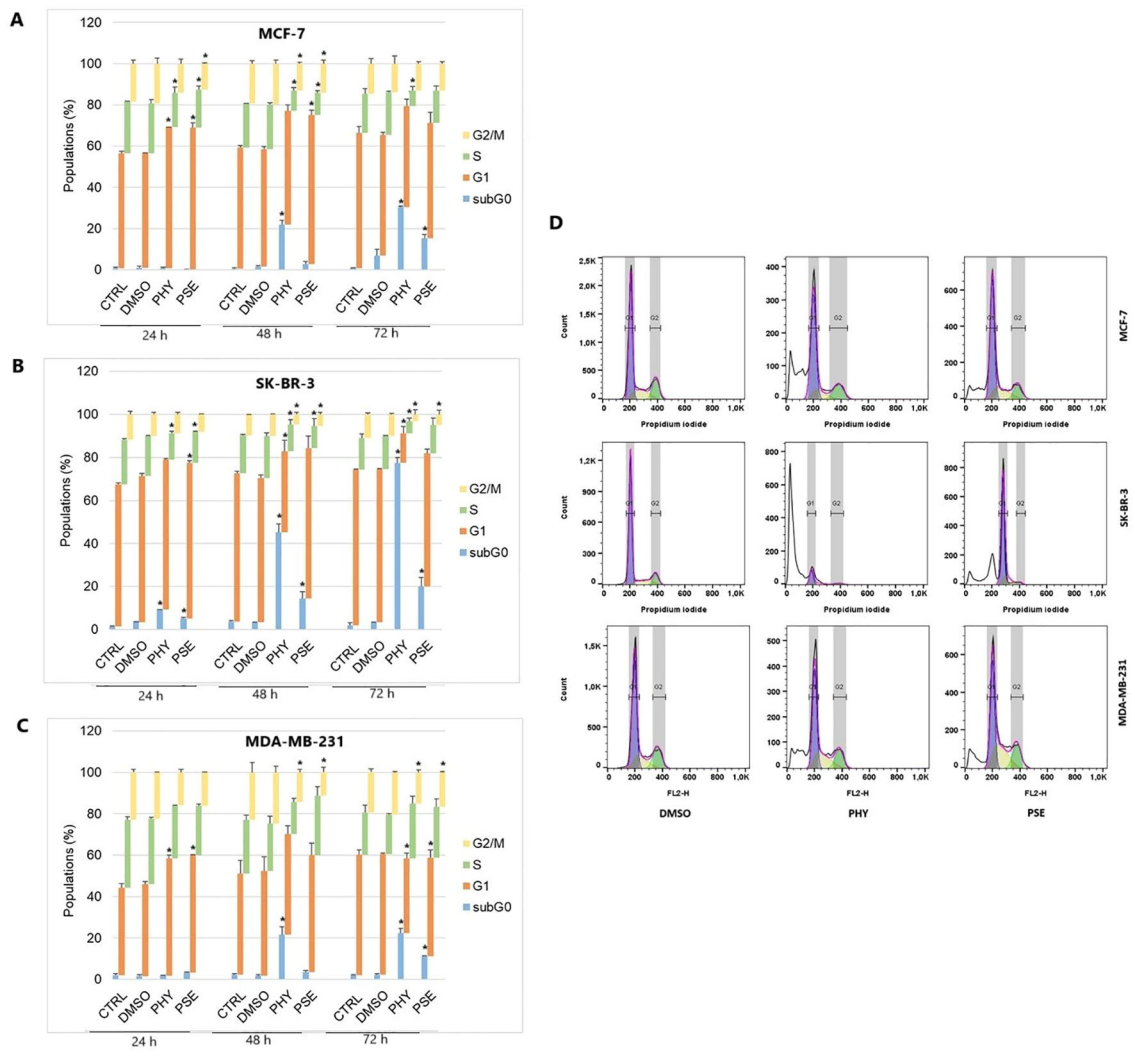
Cell cycle distribution of MCF-7 **(A)**, SK-BR-3 **(B)**, and MDA-MB-231 **(C)** cells treated with IC_50_ of PHY/PSE for 24, 48, and 72 (h) Representative diagrams illustrating cell cycle distribution of BC cells treated with IC_50_ of PHY and PSE for 72 h **(D)**. Results are presented as a mean ± standard deviation of independent experiments (*p < 0.05 compared to DMSO control, based on ordinary one-way ANOVA with Dunnett's *post hoc* test).

To investigate whether cell cycle arrest contributed to the molecular mechanism of the antiproliferative response mediated by PSE and PHY, we performed flow cytometry analysis (**Figures 3**, **Supplementary Figure S1**-**Supplementary Figure S3**). The analysis clearly showed G1 cell cycle arrest in MCF-7 and MDA-MB-231 cell lines at 24 h of PHY treatment, with an increase in the percentage of cells in the G1 phase of approximately 13% (MCF-7) and 12.1% (MDA-MB-231), respectively. At 48 h of PHY exposure, we observed the disappearance of the cell cycle arrest and a time-dependent accumulation of PHY-treated MCF-7 and MDA-MB-231 cells in the SubG0/G1 phase (cells with fragmented DNA), which is a typical feature of apoptosis. On the other hand, PSE treatment resulted in a longer arrest, observed at 24 h, which persisted up to 48 h in MCF-7 cells, with an increase in the relative percentage of cells in the G1 phase of 13.3% at 24 h and 15.6% at 48 h of treatment. However, in MDA-MB-231 cells, the effect of PSE on cell cycle distribution was similar to that of PHY, since G1 cell cycle arrest was observed only at 24 h of the treatment, with an increase in cells in the G1 phase of approximately 12.2%. Interestingly, in SK-BR-3 cells, PSE treatment showed only a weak cell cycle arrest at 24 h, with approximately 4.3% of cells in the G1 phase. Furthermore, PHY did not show significant changes in the number of cells in the G1 phase of the cell cycle, with approximately 1.8% of cells.

**Figure 4:**
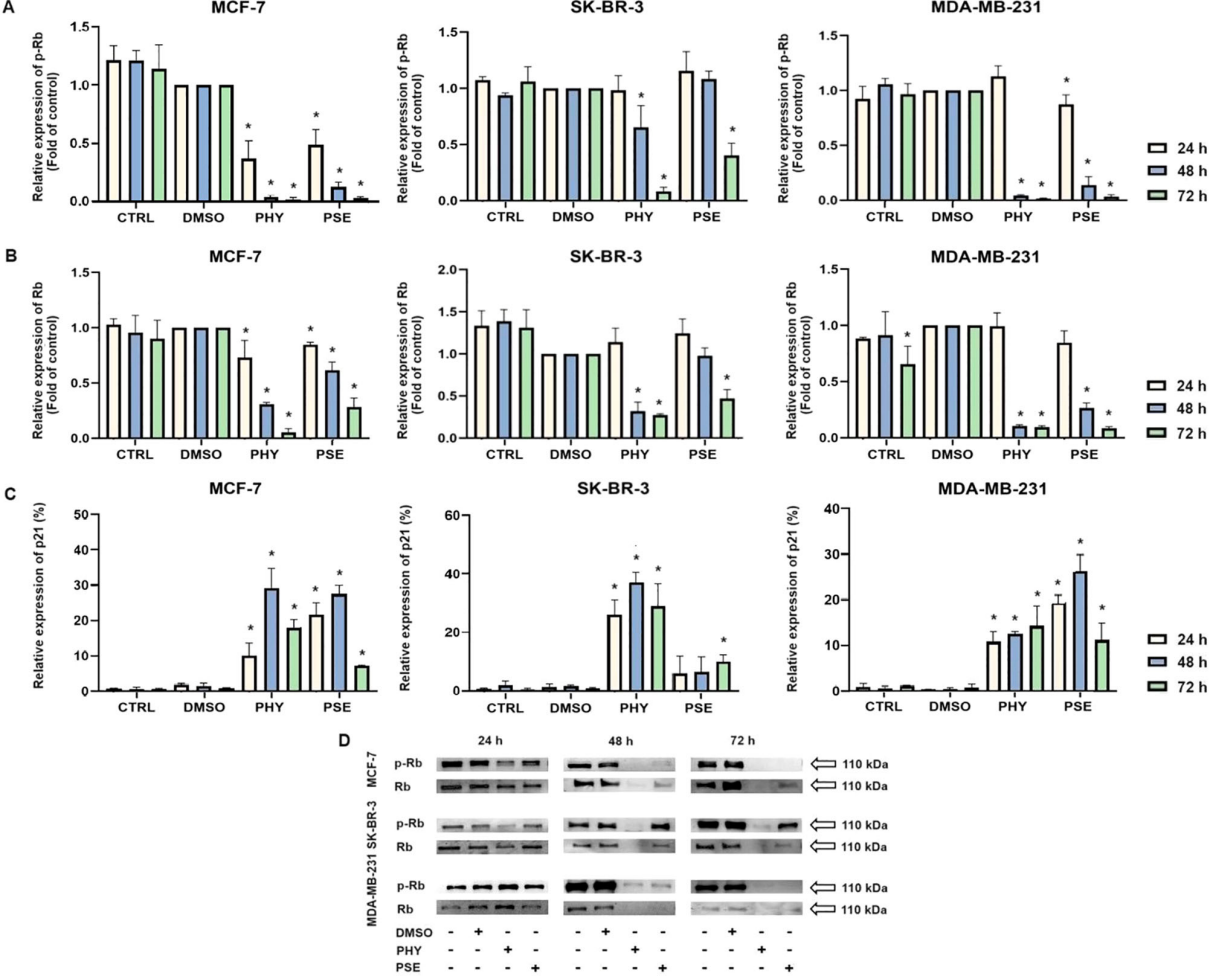
Western blot analyses of Rb and phospho-Rb protein expression in MCF-7, SK-BR-3, and MDA-MB-231 cell lines **(A, B, D)**. Flow cytometry analysis of p21 expression **(C)**. Cells were treated with IC_50_ of PHY/PSE for 24, 48, and 72 h. Relative protein levels are expressed as mean ± standard deviation of independent experiments. (*p < 0.05 compared to DMSO control, based on ordinary one-way ANOVA with Dunnett's *post hoc* test).

For further investigation of the molecular mechanism of G1 checkpoint arrest, we analysed the expression of proteins involved in cell cycle progression. Our results suggest the impact of PSE and PHY on the expression of cell cycle regulatory proteins, p21, phospho-Rb, and Rb. The results clearly show a time-dependent upregulation in the expression of p21 (**Figure 4C**) in PSE- and PHY-treated BC cells.

Moreover, significant time-dependent downregulation was detected in the expression of phosphorylated Rb (**Figures 4A, D**), while the best effect was observed in the MCF-7 cell line, followed by MDA-MB-231 and SK-BR-3 cells. Additionally, total Rb protein levels were evaluated (**Figures 4B, D**), and a significant time-dependent decrease in Rb expression was observed in both PSE- and PHY-treated BC cell lines, probably due to a loss of a cell cycle arrest and accumulation of cells in either early or late stages of apoptosis. However, at 24 h after treatment, no significant changes in protein levels of total Rb were observed in the MDA-MB-231 and SK-BR-3 cell lines. Taken together, the results of protein level changes were consistent with those of the cell cycle distribution analysis and indicated G1 cell cycle arrest of PSE/PHY treatment.

### 3.3 PSE- and PHY-mediated apoptotic death analysis

Based on the results above, we performed further analyses to investigate the molecular mechanisms of PSE-/PHY-initiated programmed cell death.

#### 3.3.1 Annexin V/PI staining

**Figure 5:**
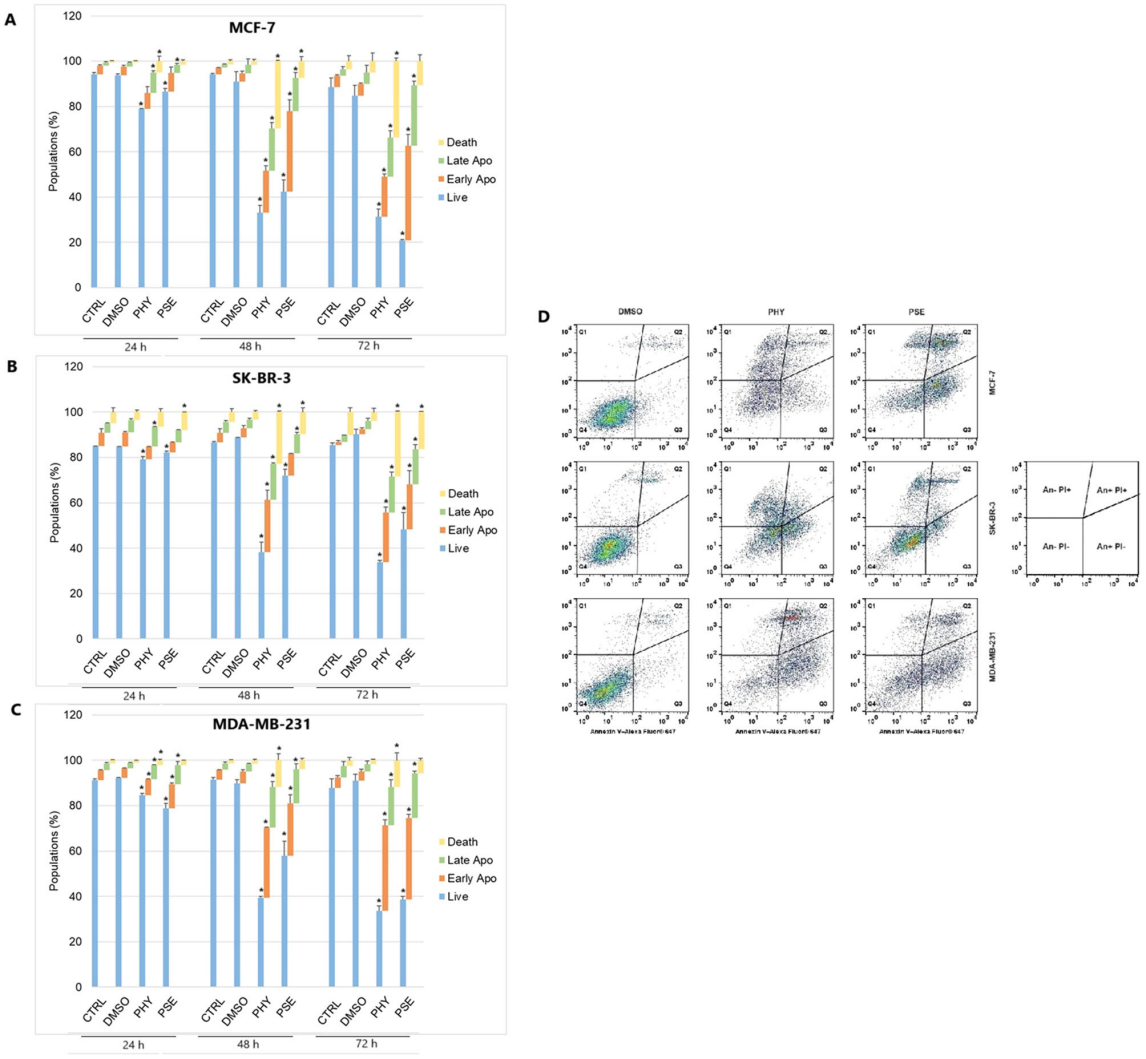
Annexin V/propidium iodide double staining of MCF-7 **(A)**, SK-BR-3 **(B)**, and MDA-MB-231 **(C)** cell lines treated with IC_50_ of PHY/PSE for 24, 48, and 72 (h) Representative dot plots illustrating apoptosis occurrence using Annexin V/PI staining in BC cells incubated with IC_50_ of PHY and PSE for 72 h **(D)**. Live cells (An−PI−), early apoptotic (An+PI−), late apoptotic (An+PI+), and dead cells (An−PI+) in a cell population. Results are presented as mean ± standard deviation of independent experiments (*p < 0.05 compared to DMSO control, based on ordinary one-way ANOVA with Dunnett's *post hoc* test).

A significant time-dependent increase was observed in early and late apoptotic as well as dead cell populations, with a subsequent decrease in the live cell population in PSE-/PHY-treated MCF-7, SK-BR-3, and MDA-MB-231 cells (**Figures 5A–D**, **Supplementary Figure S4**-**Supplementary Figure S6**). Analysis showed a significant increase in early apoptotic MCF-7 after 48 and 72 h of treatment with PSE/PHY, while the percentage of cells in the late apoptosis stage was significantly increased at all exposure times (24, 48, and 72 h). The relative percentage of the apoptotic cell population after 24, 48, and 72 h of treatment was determined to be 16.1%, 37.2%, and 35.0% for PHY, and 11.8%, 50.1%, and 68.4% for PSE, respectively. Similarly, PSE treatment resulted in an increase of Annexin-positive, propidium iodide-negative SK-BR-3 cells at 48 and 72 h; however, PHY showed a significant increase only at 48 h of the treatment. Moreover, the number of An+PI+-stained SK-BR-3 cells was increased after 48 and 72 h of treatment with PSE, while the PHY treatment resulted in an increase after all exposure times. The relative percentage of apoptotic cells after 24, 48, and 72 h of treatment was determined to be 14.2%, 39.0%, and 37.7% for PHY, and 9.7%, 18.2%, and 35.5% for PSE, respectively. Additionally, in the MDA-MB-231 cell line, an increase in the early apoptotic as well as late apoptotic cell population was observed after 24, 48, and 72 h of treatment with both tested substances. The relative percentage of the apoptotic cell population after 24, 48, and 72 h of treatment was determined to 13.2%, 48.7%, and 54.6% for PHY, and 19.1%, 38.0%, and 55.5% for PSE, respectively. These results suggest that the pro-apoptotic activity of PSE/PHY varied between *in vitro* models of individual molecular subtypes of BC in a time-dependent manner.

#### 3.3.2 Detection of mitochondrial membrane potential changes

The potential disruption of mitochondrial functional integrity was evaluated using TMRE and JC-1 fluorescent probes. The accumulation of TMRE dye in PSE- and PHY-treated BC cell lines was significantly reduced (**Supplementary Figures S7**-**Supplementary Figure S9**) indicating a reduction of mitochondrial membrane potential in treated cells. Moreover, statistics show a stronger effect of PHY compared to PSE in each BC cell line. The relative percentage of cells with decreased MMP is presented in **Supplementary Table S2**.

**Figure 6:**
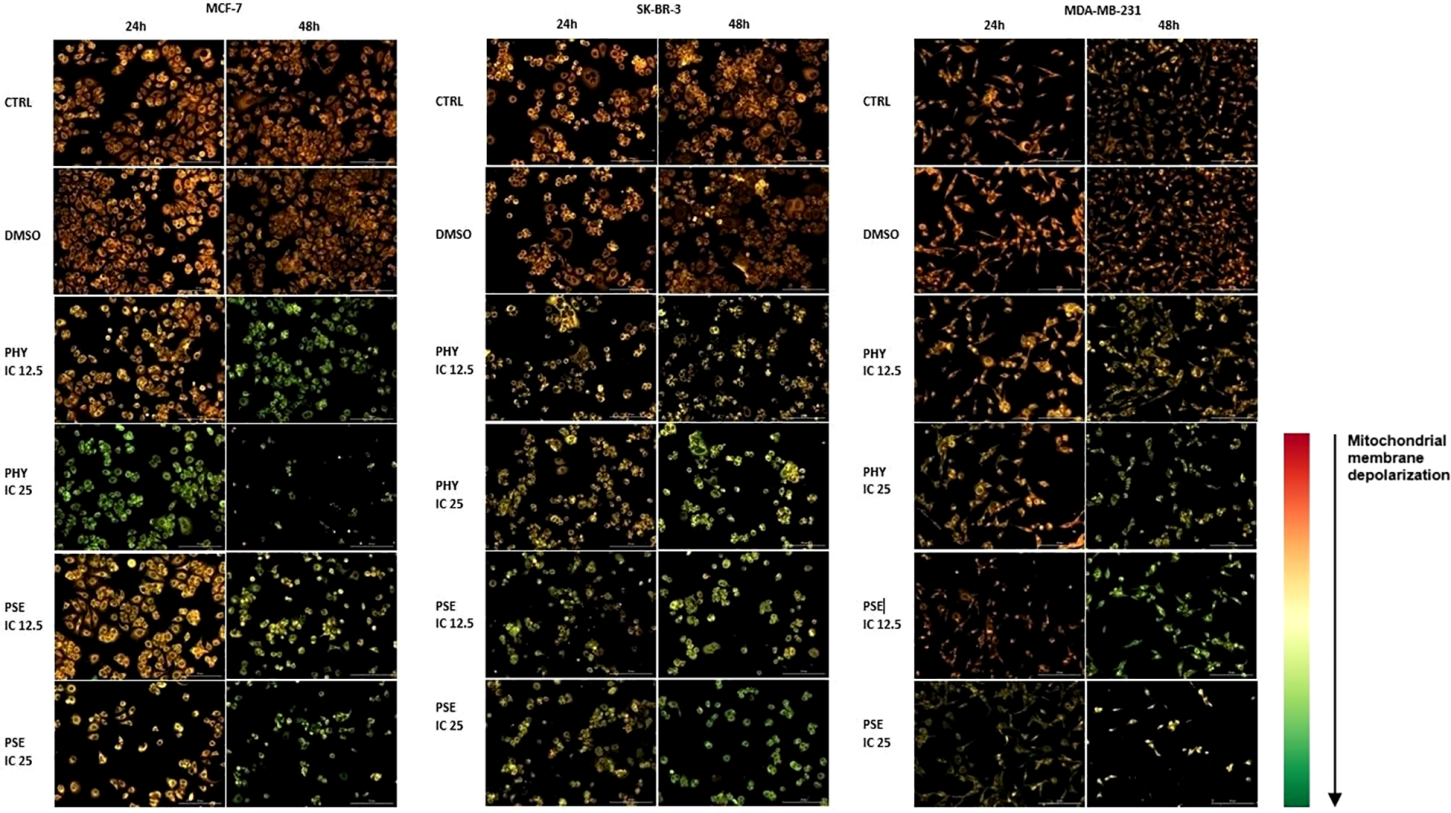
Representative fluorescence microscopic images demonstrating disruption of mitochondria function in PHY- and PSE-treated MCF-7, SK-BR-3, and MDA-MB-231 cell lines. Cells were incubated with IC_12.5_, IC_25_, and IC_50_ values of tested compounds for 24 and 48 h. Treatment with IC_50_ of PHY/PSE caused cell detachment of treated groups and therefore was excluded from the experiment. Changes in mitochondrial membrane potential are visualised using JC-1 fluorescent probe. Scale bar = 200 µm.

Additionally, changes in mitochondria function were detected using a JC-1 fluorescent probe. As presented in **Figure 6**, the control and DMSO-treated group showed relatively high MMP levels; however, administration of PSE and PHY resulted in a decrease in MMP. In JC-1 analysis, treatment with IC_50_ of PSE/PHY caused cell detachment of treated groups and therefore was excluded from the experiment.

#### 3.3.3 Modulation of Bcl-2 family protein expression

**Figure 7:**
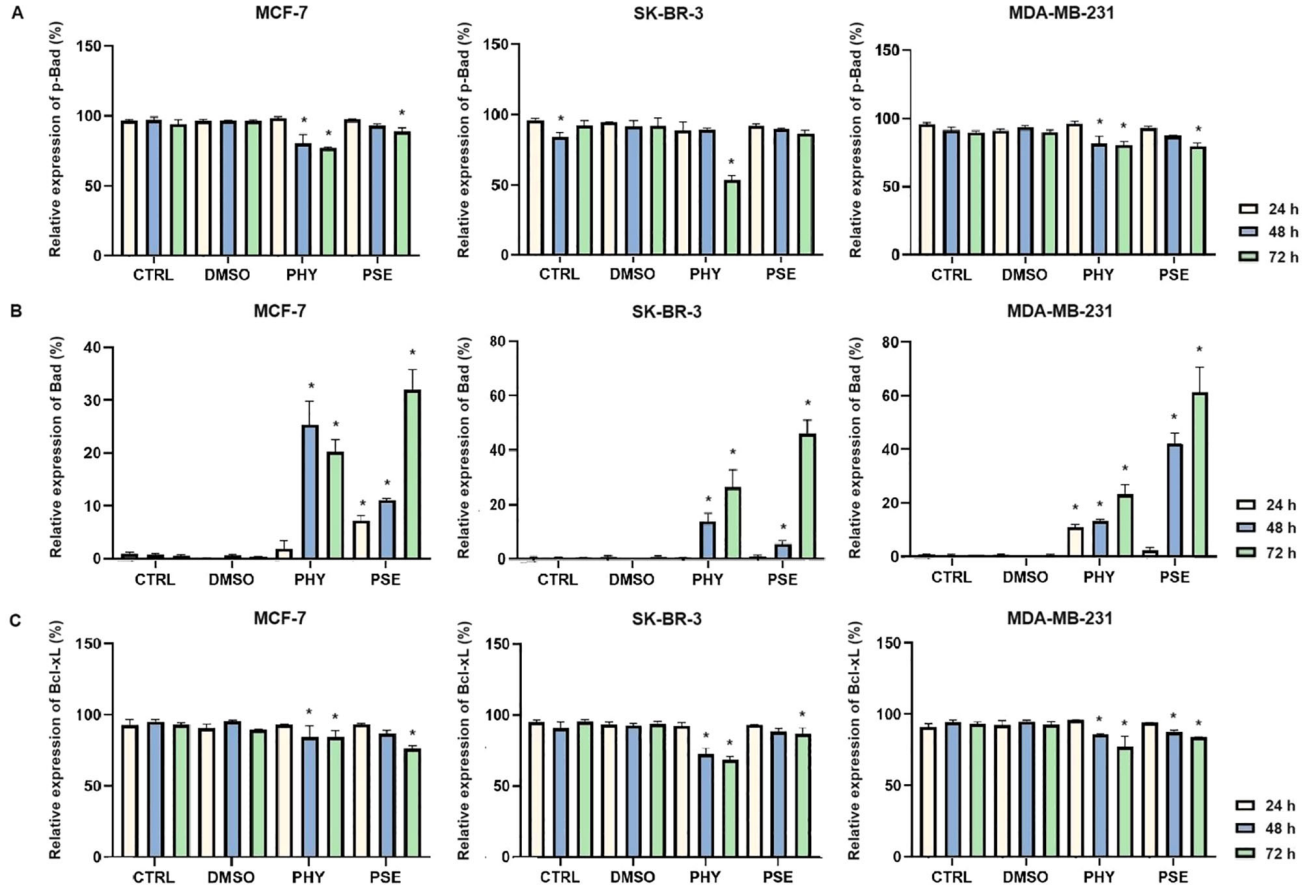
PHY- and PSE-mediated changes in the expression of Bcl-2 family proteins—p-Bad **(A)**, Bad **(B)**, Bcl-xL **(C)**. MCF-7, SK-BR-3, and MDA-MB-231 cells were treated with IC_50_ of PHY and PSE for 24, 48, and 72 (h) Results are presented as mean ± standard deviation of independent experiments (*p < 0.05 compared to DMSO control, based on ordinary one-way ANOVA with Dunnett's *post hoc* test).

Our results, based on flow cytometry data, suggest a significant time-dependent upregulation in total Bad expression (**Figure 7B**) in PSE- and PHY-treated BC cells, with concurrent downregulation in the protein level of the inactive, phosphorylated form of Bad (**Figure 7A**). Furthermore, the expression of the anti-apoptotic protein Bcl-xL significantly decreased in a time-dependent manner (**Figure 7C**). Notably, upregulated ratio Bad/Bcl-xL suggests PSE-/PHY-mediated activation of mitochondrial apoptotic pathway in BC cells.

#### 3.3.4 Analysis of cytochrome *c* release, caspase-9 cleavage, activation of executioner caspases-3 and -7, and PARP cleavage

**Figure 8:**
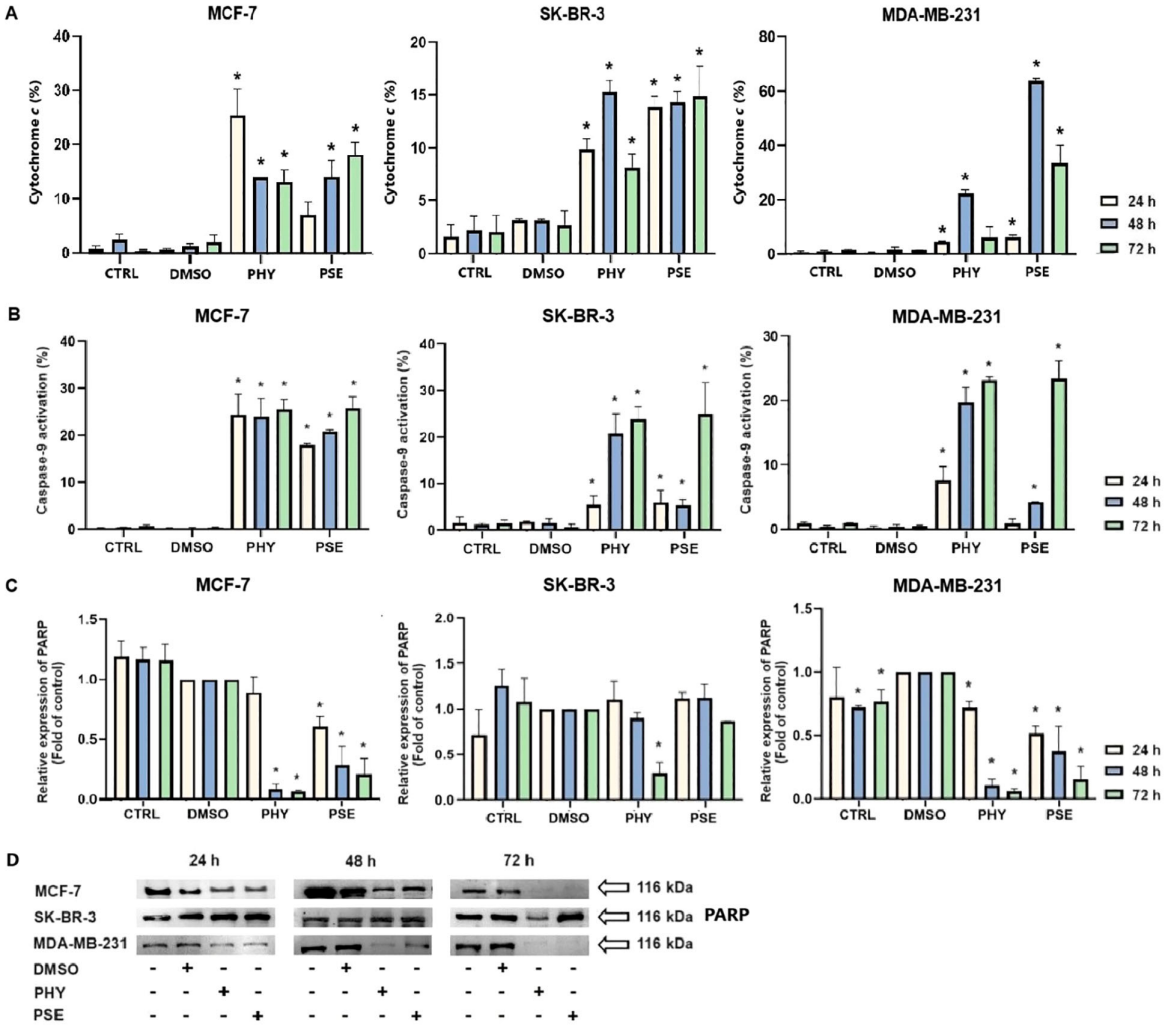
Relative percentage of cells positive for cytochrome *c* released into the cytoplasm **(A)**. PHY- and PSE-mediated caspase-9 cleavage **(B)**. Western blot analysis of PARP cleavage **(C, D)**. Cells were treated with IC_50_ of PHY/PSE for 24, 48, and 72 (h) Results are presented as mean ± standard deviation of independent experiments (*p < 0.05 compared to DMSO control, based on ordinary one-way ANOVA with Dunnett's *post hoc* test).

Significant time-dependent release of cytochrome *c* was observed from intermembrane space into the cytosol in PSE-/PHY-treated BC cells (**Figure 8A**). In the PHY-treated MCF-7 cell line, maximum increase was observed at 24 h, with 25.5 ± 3.36% cells positive for cytochrome *c.* However, SK-BR-3 an MDA-MB-231 cells showed delayed effects, and maximal increase was observed at 48 h, with 15.3 ± 0.86% and 22.3 ± 1.07% positivity, respectively. On the other hand, treatment with PSE showed an increase in cytochrome *c* positivity at 72 h in MCF-7 and 48 h in MDA-MB-231 cells, with 18.2 ± 1.1% and 63.7 ± 0.89% of cytochrome *c*-positive cells, respectively. Although PSE treatment resulted in a significant release of cytochrome *c* in the SK-BR-3 cell line, no time-significant differences were observed.

Moreover, PSE and PHY were able to induce caspase-9 cleavage in a time-dependent manner (**Figure 8B**). PHY was more efficient, compared to PSE, while the strongest effects were observed in the MCF-7 cell line, with a significant increase in caspase-9 activation as early as 24 h after treatment. In SK-BR-3 cells, delayed effects were observed, with a significant increase in caspase-9 activation after 48 h of PHY treatment and 72 h of PSE treatment. PSE-/PHY-treated MDA-MB-231 cells showed a delayed caspase-9 cleavage at 48 h of treatment.

**Figure 9:**
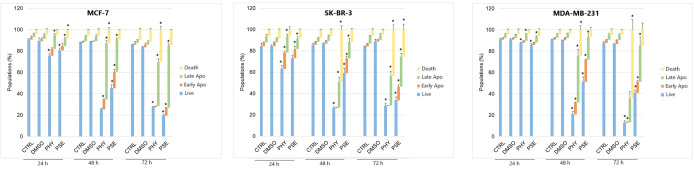
Relative percentage of cells positive for caspase-3/-7 and/or SYTOX™ AADvanced™. Cells were treated with IC_50_ of PHY/PSE for 24, 48, and 72 h. Results are presented as mean ± standard deviation of independent experiments (*p < 0.05 compared to DMSO control, based on ordinary one-way ANOVA with Dunnett's *post hoc* test).

Furthermore, subsequent activation of executioner caspases-3 and -7 was evaluated. The results show a significant time-dependent increase in the caspase-3/-7 activity after PSE/PHY treatment (**Figure 9**). The highest increase in caspase activity was noticed at 48 h, in MCF-7 (approximately 62%; early + late apoptosis) and MDA-MB-231 (approximately 55%) cells treated with PHY. The relative percentage of early apoptotic cells was determined to 9.2 ± 0.6% and 11.1 ± 0.4%, respectively. Interestingly, in SK-BR-3 cells, maximal increase was detected at 24 h (approximately 31%); however, no time-significant differences in the percentage of caspase-positive cells were observed. Treatment with PSE resulted in the strongest effect on caspase activation at 72 h in MCF-7 (approximately 66%), MDA-MB-231 (approximately 45%), and SK-BR-3 (approximately 41%) cells, with the relative percentage of early apoptotic cells determined as 7.7 ± 0.2 for MCF-7, 40.7 ± 2.9 for MDA-MB-231, and 13.0 ± 1.7 for SK-BR-3.

Moreover, PSE/PHY treatment resulted in a PARP cleavage, which was found to be time dependent (**Figures 8C, D**). The strongest effect was observed in PHY-treated cells in comparison to PSE treatment. Additionally, among all three *in vitro* models, comparable effects were determined in MCF-7 and MDA-MB-231 cells, while SK-BR-3 seemed to be the most resistant to PSE/PHY treatment, with a significant decrease in protein expression observed only at 72 h in PHY-treated cells.

### 3.4 PSE- and PHY-mediated oxidative stress

Further experiments were focused on the involvement of oxidative/nitrosative stress and the modulation of the cell antioxidant enzyme system in the mechanism of PSE- and PHY-mediated apoptosis.

#### 3.4.1 Analysis of ROS and RNS levels in PSE/PHY-treated cells

**Figure 10:**
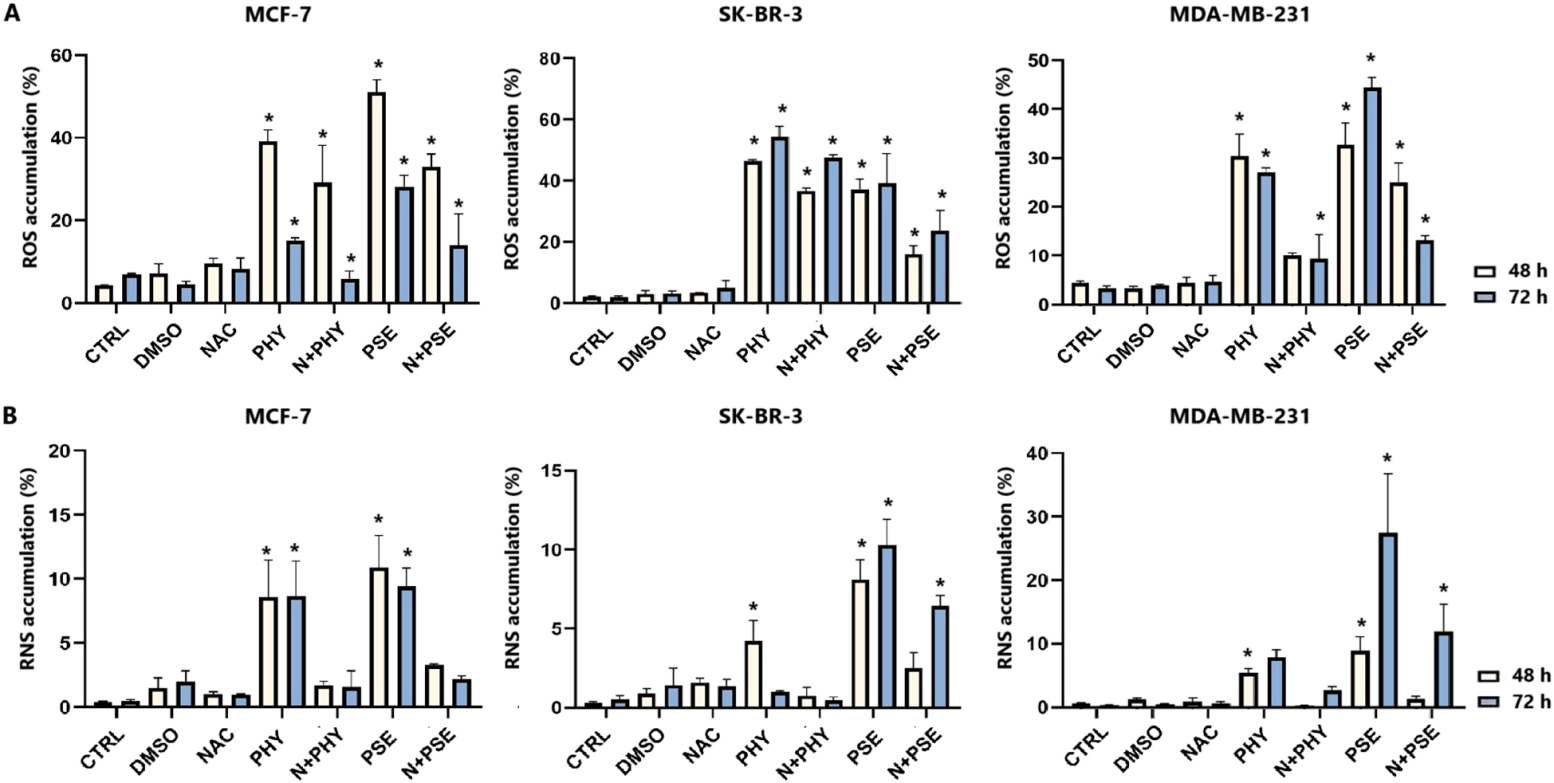
Relative percentage of cells positive for superoxide **(A)**. Relative percentage of cells positive for nitric oxide **(B)**. Cells were treated with IC_50_ of PHY/PSE for 48 and 72 **(h)** Results are presented as mean ± standard deviation of independent experiments (*p < 0.05 compared to DMSO control, based on ordinary one-way ANOVA with Dunnett's *post hoc* test).

A significant increase in superoxide radical levels was observed in both PSE- and PHY-treated BC cell lines (**Figure 10A**, **Supplementary Table S3**). In MCF-7 cells, the strongest effects were observed at 48 h of PSE/PHY treatment. SK-BR-3 cells showed slightly delayed effects, with a maximum at 72 h of treatment. In the MDA-MB-231 cell line, the strongest effect of PHY was observed at 48 h, while the effect of PSE was the most potent at 72 h of treatment. These results suggest that the pro-oxidative effects of PSE and PHY appear to be time and cell line dependent. Moreover, N-acetylcysteine (NAC) was used as an antioxidant, and it was capable of scavenging superoxide anions in treated cells, partially.

Furthermore, PSE/PHY was able to induce RNS production in all tested BC cell lines in a time-dependent manner (**Figure 10B**, **Supplementary Table S4**). PSE showed higher efficacy compared to PHY, while the strongest effect was observed in MDA-MB-231 at 72 h of treatment.

#### 3.4.2 Analysis of the antioxidant response in PSE-/PHY-treated cells

**Figure 11:**
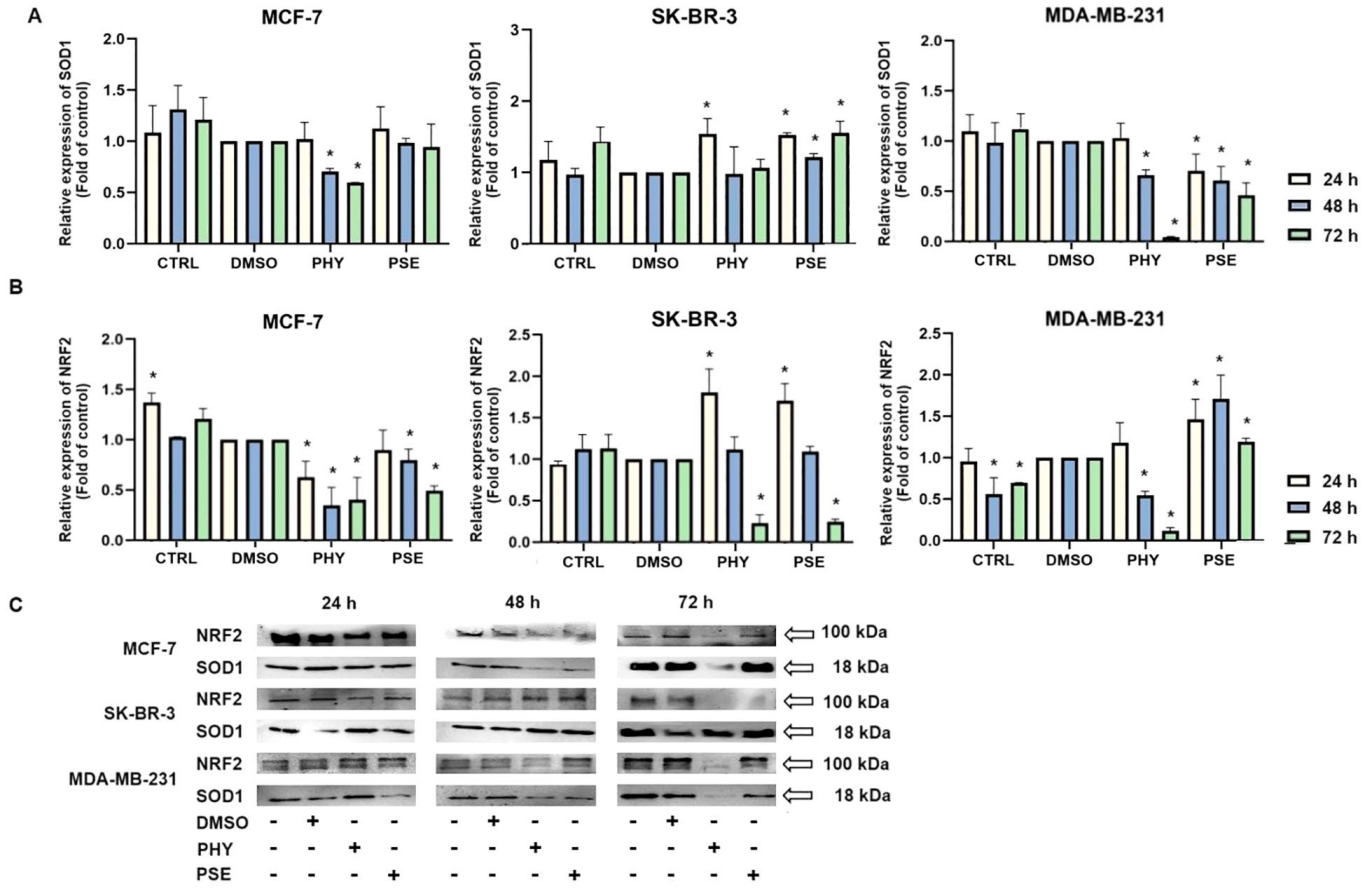
Western blot analyses of SOD1 **(A)** and NRF2 **(B)** protein expression in MCF-7, SK-BR-3, and MDA-MB-231 cell lines **(C)**. Cells were treated with IC_50_ of PHY/PSE for 24, 48, and 72 (h) Relative protein levels are expressed as mean ± standard deviation of independent experiments (*p < 0.05 compared to DMSO control, based on ordinary one-way ANOVA with Dunnett's *post hoc* test).

Currently, in our study, changes in SOD1 expression were observed in BC cells treated with PSE and PHY (**Figures 11A, C**). The strongest effect was observed in the MCF-7 cell line, with a significant decrease in protein levels at 48 and 72 h of treatment. On the contrary, SK-BR-3 cells did not show an effect of PSE/PHY on the targeting of SOD1, with an increase in protein expression. Moreover, **Figures 11B, C** show a time-dependent inhibition of NRF2 protein expression. The strongest effect was observed in the PHY-treated MCF-7 cell line, with a significant decrease at 24, 48, and 72 h of treatment. PSE showed lower efficacy compared to PHY. These results suggest the involvement of oxidative/nitrosative stress in PSE- and PHY-induced apoptosis in BC cell lines. Moreover, inhibition of the antioxidant response through modulation of SOD1 expression was demonstrated for the first time in BC models.

### 3.5 Analysis of PSE-/PHY-mediated DNA damage induction and DNA damage repair inhibition

**Figure 12:**
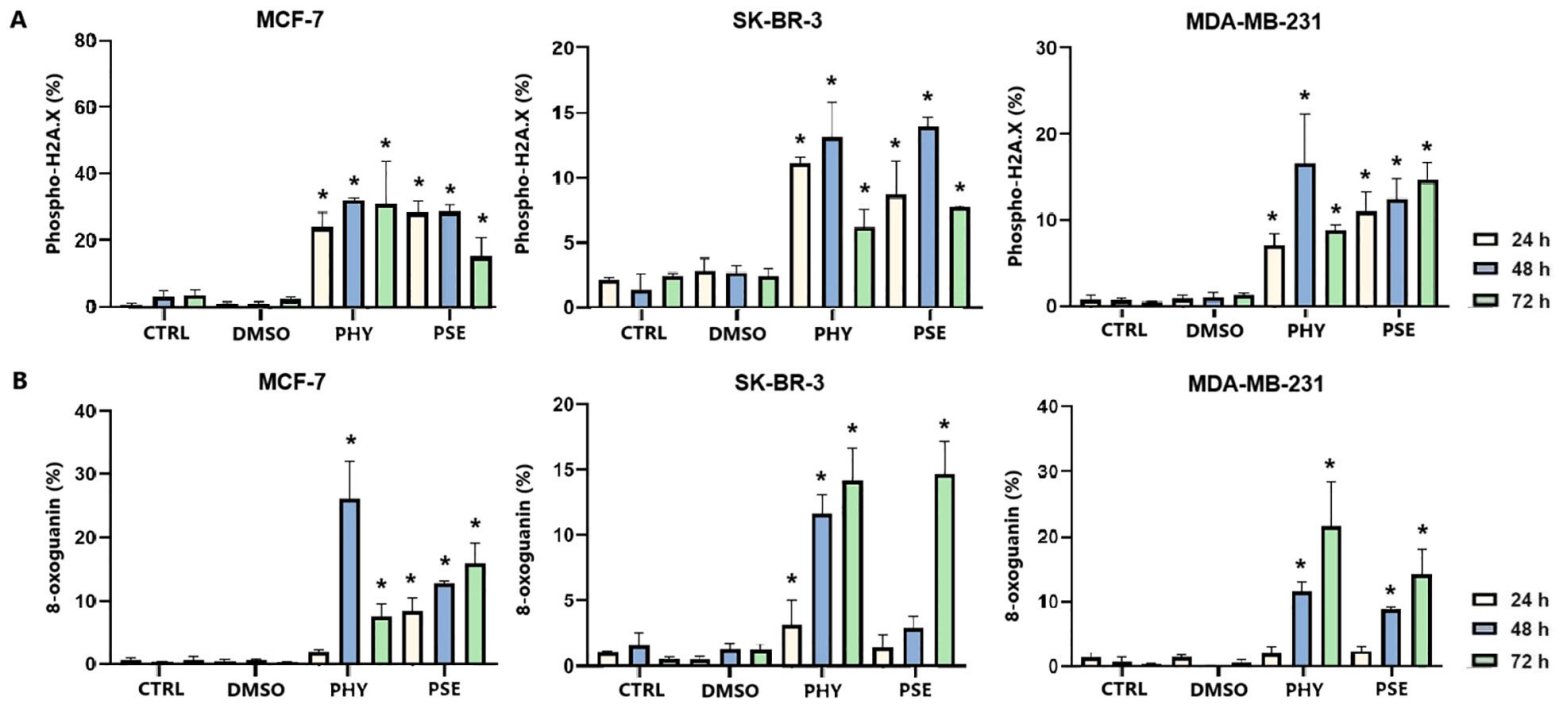
Relative percentage of cells positive to phospho-H2A.X **(A)** and 8-oxoguanine **(B)**. Cells were treated with IC_50_ of PHY/PSE for 24, 48, and 72 h. Results are presented as mean ± standard deviation of independent experiments (*p < 0.05 compared to DMSO control, based on ordinary one-way ANOVA with Dunnett's *post hoc* test).

**Figure 13:**
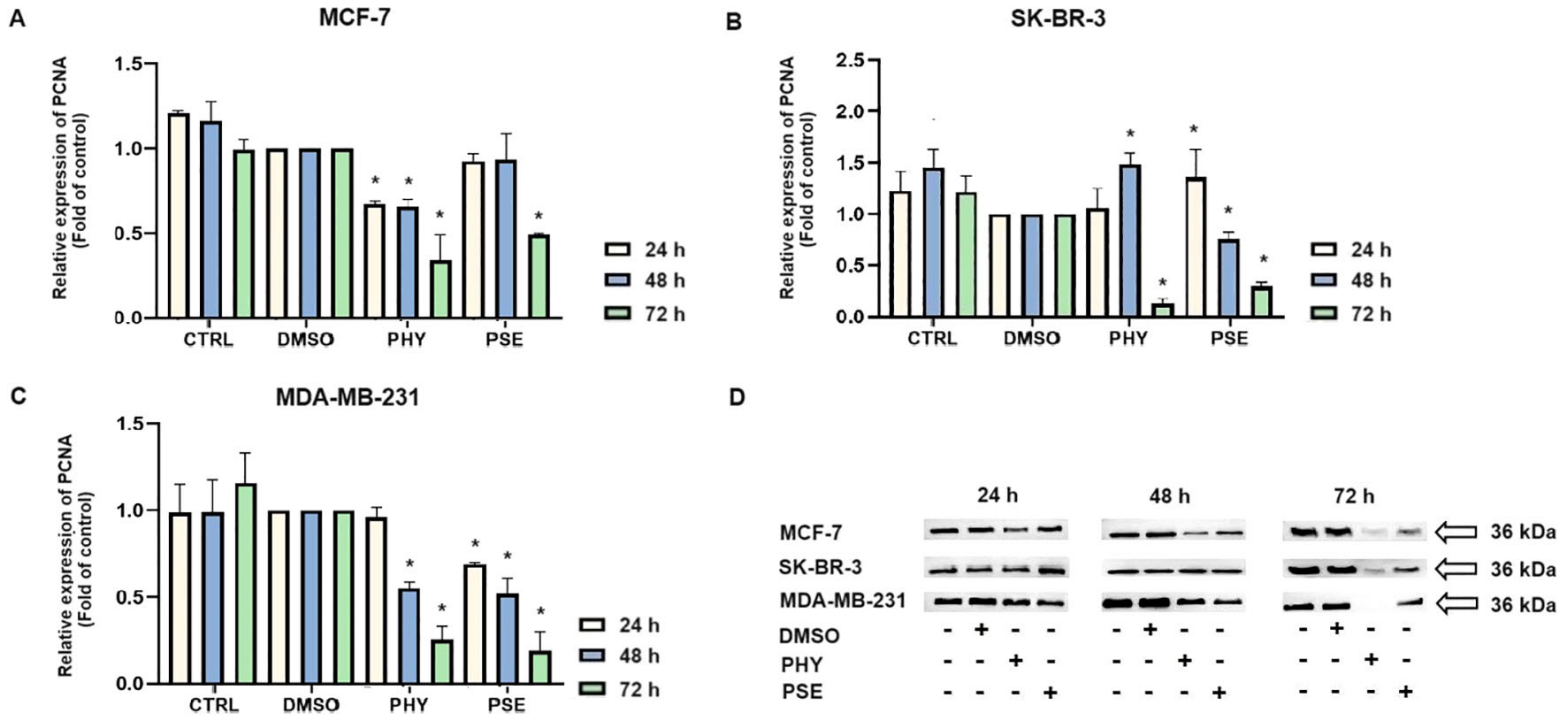
Western blot analyses of PCNA expression in MCF-7, SK-BR-3, and MDA-MB-231 cell lines **(A–D)**. Cells were treated with IC_50_ of PHY/PSE for 24, 48, and 72 h. Relative protein levels are expressed as mean ± standard deviation of independent experiments (*p < 0.05 compared to DMSO control, based on ordinary one-way ANOVA with Dunnett's *post hoc* test).

The induction of DNA damage was evaluated by measuring the levels of 8-oxoguanine, oxidation product of guanine, phosphorylated histone H2A.X, and the DNA repair-related protein PCNA. Treatment with PSE and PHY resulted in a time-dependent increase in the levels of phosphorylated histone H2A.X (**Figure 12A**), the downstream target of ATM kinase, indicating that treatment caused double-strand breaks in the DNA. The strongest effects were observed in MCF-7 cells, followed by the MDA-MB-231 and SK-BR-3 cell lines. PSE appeared to be slightly less potent than PHY. Additionally, both were able to elevate 8-oxoguanine levels, which is the main mutagenic oxidative damage product. The results suggest a higher efficacy in PHY-treated BC cells compared to PSE treatment (**Figure 12B**). Furthermore, PSE and PHY were able to modulate PCNA expression, which plays a role in DNA replication and repair. The strongest effect was determined in MCF-7 (**Figures 13A, D**) and MDA-MB-231 cells (**Figures 13C, D**), while the SK-BR-3 (**Figures 13B, D**) cell line was the most resistant.

### 3.6 PSE-/PHY-mediated inhibition of PD-1/PD-L1 immune checkpoint

**Figure 14:**
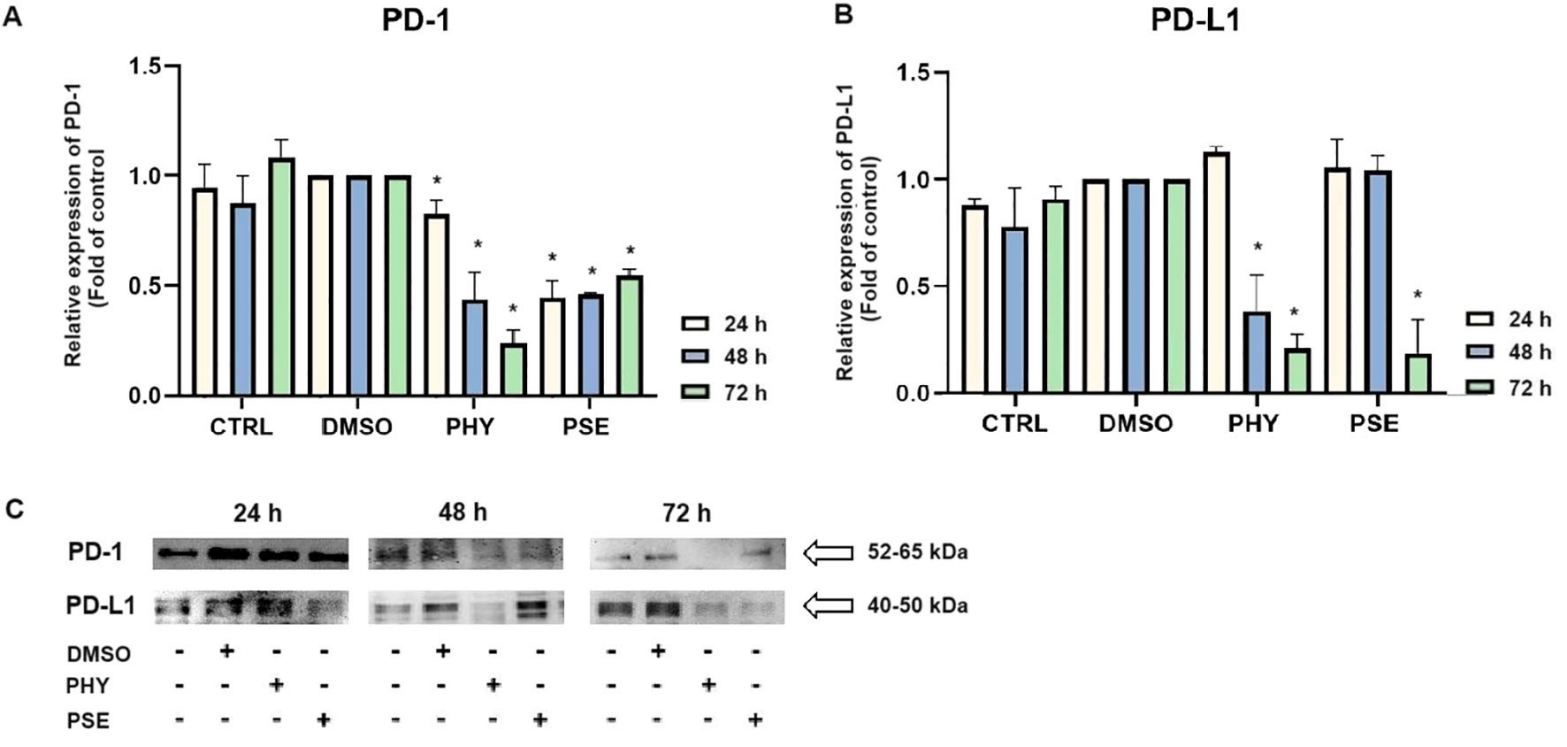
Western blot analyses of PD-1 and PD-L1 expression in MCF-7, SK-BR-3, and MDA-MB-231 cell lines **(A–C)**. Cells were treated with IC_50_ of PHY/PSE for 24, 48, and 72 h. Relative protein levels are expressed as mean ± standard deviation of independent experiments (*p < 0.05 compared to DMSO control, based on ordinary one-way ANOVA with Dunnett's *post hoc* test).

Here, we demonstrate for the first time that PSE- and PHY-mediated time-dependent downregulation of the PD-1 (**Figures 14A, C**) and PD-L1 (**Figures 14B, C**) expression occurs in the TNBC model, represented by the MDA-MB-231 cell line.

## 4 Discussion

Despite progress in diagnostics and treatment, BC remains a leading cause of death, with high recurrence and resistance to chemotherapy. This has driven interest in alternative therapies like natural compounds with anticancer effects. Lichens, known for their cytotoxic and multitarget actions, are one such source (6). The potential antiproliferative, pro-apoptotic, antimigratory, and TME-modulating activities of PSE and PHY have been described in several *in vitro* cancer models (5, 8, 10, 17). However, their role in BC remains underexplored. Our study investigated their molecular effects in BC cell lines (MCF-7, SK-BR-3, MDA-MB-231), showing time- and dose-dependent inhibition of cell proliferation. PSE proved more effective than PHY, likely due to synergistic interactions among its components. Our findings align with previous studies demonstrating the anticancer potential of *P. furfuracea* extracts and PHY. For example, Kello et al. (2023) (8) evaluated PSE-/PHY-mediated inhibition of metabolic activity in a broad spectrum of *in vitro* cancer models, including cervical, colon, breast, lung, melanoma, leukaemia, and ovarian cancer. Similarly, a separate study showed antiproliferative effects of PSE/PHY in Jurkat cells (5) and the effect of PHY in LNCap and e2f cells (18).

Beyond antiproliferative effects, we evaluated whether PSE and PHY induce cell cycle arrest. Lichen-derived compounds often trigger arrest at G0/G1, S, or G2/M phases (5, 6, 19). Our flow cytometry results showed that both PSE and PHY induced G1 phase arrest in MCF-7 and MDA-MB-231 cells after 24 h, while only PSE caused mild G1 arrest in SK-BR-3 cells. G1–S transition is driven by cyclin–CDK complexes that phosphorylate Rb, releasing E2F and promoting cell cycle progression. This is counteracted by CDK inhibitors, like p21, which is regulated by p53 (20–23). We observed time-dependent upregulation of p21 and downregulation of phosphorylated Rb in all BC cell lines tested, especially MCF-7, aligning with G1 arrest at 24–48 h. Total Rb also declined by 48–72 h suggesting progression from arrest to apoptosis. This is the first report, to our knowledge, of PSE- and PHY-induced G1 cell cycle arrest and modulation of regulatory proteins across three *in vitro* BC models. Our data suggest that G1 arrest may be subtype dependent. Supporting this, usenamine A, a lichen-derived compound, induced G2/M arrest in MDA-MB-231 by downregulating Cyclin B1, Cyclin A, and CDK2 (19). In our previous work on leukaemia cells, PHY induced G1 arrest in Jurkat cells, while PSE caused S-phase arrest—both linked to decreased phosphorylated Rb and increased p21 expression, consistent with current findings (5). In contrast, other studies reported increased sub-G0/G1 populations in melanoma and colon cancer cells treated with PSE/PHY, but without cell cycle arrest. These results highlight a tissue-specific response to PSE and PHY, with arrest occurring at G1, S, or G2/M depending on cancer type (24).

Phosphatidylserine (PS) externalisation is an early marker of apoptosis, reflecting membrane changes (25, 26). In our study, PSE and PHY treatment led to a time-dependent rise in apoptotic and dead BC cells, peaking at 48–72 h, with a decline in viable cells. This supports earlier findings on the proapoptotic effects of *P. furfuracea* and PHY in various cancer models (5, 7, 17, 27). Our previous work in Jurkat cells showed PS externalisation, with PSE being more potent than PHY (5). Moreover, Šeklić et al. (2018) (28) reported apoptosis induction by *P. furfuracea* extracts in colon cancer cells, with extract efficacy varying by solvent and cell line. Annexin V assays also confirmed PHY's proapoptotic activity in HCT-116, DLD-1, and HaCaT cells (29). These results suggest that early PS externalisation (by 24 h) is linked to G1 arrest reflecting a critical point for repair or commitment to cell death.

Apoptosis is closely linked to oxidative stress and mitochondrial dysfunction, including increased membrane permeability and loss of mitochondrial membrane potential (MMP). For the first time, we report that PSE and PHY cause time-dependent MMP disruption in BC *in vitro* models, with MMP loss increasing over time and persisting up to 72 h. This was accompanied by increased apoptosis, PS externalisation, caspase activation, and PARP cleavage. Our previous work in Jurkat cells showed similar results, with PSE having a stronger effect than PHY (5). Goga et al. (2019) (30) also observed mitochondrial dysfunction in gyrophoric acid-treated HeLa cells, where antioxidant pre-treatment reduced apoptosis pointing to oxidative stress involvement. Likewise, Kumar et al. (2020) (31) reported that usnic acid disrupted MMP and induced apoptosis *via* Bax : Bcl-2 imbalance and caspase activation in gastric cancer cells. Loss of mitochondrial membrane potential (MMP) reflects outer membrane permeabilisation, regulated by Bcl-2 family proteins—pro-apoptotic (Bad, Bax, Bak, Bid) and anti-apoptotic (Bcl-2, Bcl-xL, Bcl-W) (32, 33). Our flow cytometry data showed that PSE and PHY modulate these proteins in BC cells. Specifically, Bad expression increased over time in all cell lines, while its inactive phosphorylated form and anti-apoptotic Bcl-xL were downregulated by 48–72 h. These findings align with prior studies, such as that of Cardile et al. (2022) (18), that reported a pro-apoptotic Bax/Bcl-2 shift in PHY-treated prostate cancer cells. Similarly, *Physconia hokkaidensis* extract induced apoptosis in TNBC *via* caspase activation and Bcl-2 downregulation (34), and physciosporin promoted apoptosis by upregulating Bax, cleaved caspase-7, and PARP, while reducing Bcl-xL in MCF-7 and MDA-MB-231 cells (35). Mitochondrial permeabilisation leads to cytochrome *c* release, APAF-1 activation, apoptosome formation, and caspase-9 activation (36) triggering caspases-3/-7 and PARP cleavage (37). Our results suggest that PSE and PHY induce apoptosis through this mitochondrial pathway, beginning as early as 24 h and continuing through 72 h, marked by MMP loss, Bad upregulation, cytochrome *c* release, caspase activation, and PARP cleavage. Similar findings were reported in PSE-/PHY-treated Jurkat cells (5), PHY-treated A375 cells (27), and GA-treated HeLa cells (30). Oxidative stress plays a key role in cancer development by damaging proteins, lipids, and DNA through excess ROS/RNS production (38, 39). Although cancer cells already exhibit elevated ROS/RNS levels, further increases can induce cytotoxicity, cell cycle arrest, and apoptosis—making oxidative stress modulation a promising anticancer strategy (28, 40, 41). While many lichens are known for their antioxidant properties, recent studies highlight the pro-oxidant effects of lichen-derived compounds as a mechanism of cancer cell death (8, 9, 11, 42). In our study, PSE and PHY significantly increased superoxide and nitric oxide levels in BC cells from 6 h, peaking at 24 h, aligning with observed mitochondrial damage (MMP loss). Similar ROS-mediated effects were seen in PSE/PHY-treated Jurkat cells (5) and colon cancer models (28). Goga et al. (2019) reported increased ROS after GA treatment in cervical cancer cells, reversible by antioxidant pre-treatment with N-acetylcysteine (30). Likewise, *Usnea barbata* extract induced ROS and DNA damage in oral cancer cells (43). To maintain redox homeostasis, cells have evolved antioxidant defences, including scavenging molecules (e.g., glutathione, vitamins C and E) and enzymes like superoxide dismutase (SOD), catalase, and glutathione peroxidase. SOD1–3 play a key role by converting superoxide radicals into oxygen and hydrogen peroxide, which are crucial for redox balance (44). Our results showed downregulation of SOD1 in MCF-7 and MDA-MB-231 cells after 48–72 h of PSE/PHY exposure, while SK-BR-3 cells showed transient upregulation at 24 h—suggesting a differential antioxidant response. Supporting our findings, Kello et al. (2021) (5) observed decreased SOD1/2 in PSE-treated Jurkat cells.

NRF2 is a key regulator of oxidative stress, inflammation, and autophagy. Although traditionally considered a tumour suppressor, its persistent activation is now linked to tumour progression, chemoresistance, and poor prognosis (44–47). In our study, PSE and PHY caused time-dependent NRF2 deregulation in BC cells, most notably in PHY-treated MCF-7 cells. PSE also upregulated NRF2 at 24 h in MDA-MB-231 and SK-BR-3 cells potentially offering transient protection against oxidative stress—consistent with lower superoxide levels at that time. Similarly, Qi et al. (2020) (48) reported that usnic acid induced ROS and apoptosis while reducing NRF2 protein levels in lung carcinoma. Other studies have shown that lichen compounds either suppress or activate NRF2, as seen in physodic and salazinic acid-treated colorectal cancer cells, where nuclear translocation and transcript increases in NRF2 were reported (49). ROS/RNS can also cause DNA damage, activating DNA damage response (DDR) pathways and promoting apoptosis mechanisms exploited by many chemotherapeutics (30, 50, 51). In this study, PSE and PHY induced oxidative DNA damage and altered DNA repair signalling in BC cells. Both compounds increased 8-oxoguanine and phosphorylated H2A.X levels over time, especially in MCF-7 cells indicating ROS-induced double-strand breaks. These changes were accompanied by the downregulation of SOD1 and the modulation of NRF2. Additionally, PCNA expression was time-dependently reduced suggesting impaired repair capacity. These findings are in line with previous studies showing similar genotoxic effects of lichen compounds. Kello et al. (2021) observed ATM/SMC1 activation and oxidative DNA lesions in Jurkat cells after PSE/PHY treatment (5). Other reports describe DNA damage from GA in HeLa cells (30), *Usnea barbata* extract in oral cancer cells (43), and UA in gastric cancer, which upregulated DNA-PKcs, Chk-2, and p53, and phosphorylated H2A.X and ATM (31). Modulating the antitumour immune response, particularly through the PD-1/PD-L1 pathway, is a promising approach in cancer treatment (52, 53). This pathway plays a key role in immune escape, and targeting it with checkpoint inhibitors has shown promise, especially in TNBC, which has a poorer prognosis and limited treatment options (54). Our data suggest that PSE and PHY modulate PD-1 and PD-L1 expression in a time-dependent manner in a TNBC *in vitro* model. This is consistent with studies showing that UA downregulates PD-L1 in HeLa cells inhibiting proliferation, migration, and invasiveness, while enhancing T-cell cytotoxicity (55). Similarly, natural compounds, such as quercetin and resveratrol, have been shown to exert immunomodulatory effects by regulating the PD-1/PD-L1 axis and enhancing antitumour immune responses (56–59). This suggests that PSE and PHY may contribute to reshaping the tumour immune microenvironment and boosting antitumour immunity.

Our experiments demonstrated that the treatment with PSE and PHY was most effective in MCF-7 cells representing the luminal (ER+/PR+) breast cancer subtype, while the lowest sensitivity was observed in HER2-positive SK-BR-3 cells. These differences likely reflect the distinct biological characteristics of the individual BC subtypes. Luminal tumours are generally less aggressive, exhibit slower proliferation, higher differentiation status, and often retain functional cell cycle and apoptotic regulatory mechanisms (such as p21 activity or higher Bcl-2 expression). In contrast, HER2-positive cells are characterised by hyperactivation of growth and survival pathways (including PI3K/AKT and MAPK), which contributes to increased resistance to stress conditions and apoptosis induction. Therefore, it is conceivable that MCF-7 cells are more susceptible to the effects of PSE and PHY *via* modulation of the cell cycle, oxidative stress, or apoptotic pathways, whereas SK-BR-3 cells are able to better resist these effects due to activated survival mechanisms. Despite this fact, PSE and PHY were able to initiate apoptosis in SK-BR-3 cells. Some of our results thus highlight the subtype-specific response to the tested compounds, which is important to consider in further research and in the design of potential therapeutic strategies.

## 5 Conclusion

In this study, we demonstrated the antiproliferative and pro-apoptotic potential of PSE and PHY in three BC models. We confirmed that both PSE and PHY inhibited MCF-7, SK-BR-3, and MDA-MB-231 cell proliferation in a time- and dose-dependent manner. We showed that this inhibition was associated with G1 cell cycle arrest. Moreover, PSE and PHY were found to be capable of changing the redox status of BC cells. Excessive ROS/RNS production led to mitochondrial membrane damage resulting in increased permeability and release of pro-apoptotic factors, particularly cytochrome *c*, leading to the formation of an apoptosome complex with Apaf-1, which activated caspase-9 followed by cleavage of executioner caspase-3/-7. Additionally, upregulation of pro-apoptotic Bad, with concomitant downregulation of anti-apoptotic Bcl-xL protein expression was described. In this study, we also showed that oxidative stress induced DNA damage, by detection of DNA damage markers—histone H2A.X and 8-oxoguanine. Furthermore, PSE and PHY were able to modulate PCNA expression as a DNA repair marker. Finally, we also observed downregulation in the PD-1 and PD-L1 expression in the TNBC model. Taken together, the results in this paper showed a good potential of PSE and PHY as anticancer agents in BC models. We assume that PSE and PHY combined with conventional chemotherapy could enhance the efficacy of the therapy and overcome drug resistance.

## 6 Limitations and plans

The present study has some limitations that should be considered. The use of 2D *in vitro* models, although widely accepted for initial mechanistic studies, does not fully replicate the complexity of the tumour microenvironment, which may limit the translational relevance of the obtained results. Moreover, this study was focused primarily on exploring the cellular and molecular effects of *Pseudevernia furfuracea* extract (PSE) and physodic acid (PHY), without addressing aspects related to clinical applicability, such as compound production, delivery methods, dosing strategies, solubility, stability, bioavailability, and toxicity. These issues remain to be clarified in future research. Therefore, our future work will focus on validating the current findings in more physiologically relevant models, including 3D culture systems, spheroids, and *in vivo* studies. Additionally, further investigation of the pharmacokinetic properties, safety profile, and development of effective delivery systems for PSE and PHY is planned. We also aim to explore the potential synergistic effects of these compounds in combination with conventional chemotherapeutic agents to improve treatment efficacy and overcome drug resistance in BC therapy. Moreover, a detailed exploration of the mechanisms underlying their immunomodulatory effects, particularly their influence on immune checkpoint pathways (PD-1/PD-L1) in co-culture or immune-competent models, is planned.

## Data Availability

The raw data supporting the conclusions of this article will be made available by the authors, without undue reservation.
