# Supplementary material for: Antiproliferative and pro-apoptotic effects of Pseudevernia furfuracea (L.) Zopf extract and its active component physodic acid via oxidative stress and DNA damage in breast cancer cells
Source: Front Oncol. 2025 May 21;15:1557884. doi: 10.3389/fonc.2025.1557884 (PMC12133469; doi:10.3389/fonc.2025.1557884)
Supplement: Supplementary file 1 [file DataSheet1.docx]

# SUPPLEMENTARY MATERIAL

**Table S1:** List of WB antibodies

| Primary antibody | Cat. Number | Mr (kDa) | Dilution | Origin | Company |
| --- | --- | --- | --- | --- | --- |
| PARP | #9532 | 116/89 | 1:1000 | Rabbit | Cell Signalling Technology® |
| p-Rb | #72443 | 110 | 1:1000 | Rabbit |  |
| Rb | #9309 | 110 | 1:2000 | Mouse |  |
| NRF2 | #12721 | 97-100 | 1:1000 | Rabbit |  |
| PD-1 | #86163 | 52-65 | 1:1000 | Rabbit |  |
| PD-L1 | #13684 | 40-50 | 1:1000 | Rabbit |  |
| PCNA | #13110 | 36 | 1:1000 | Rabbit |  |
| SOD1 | #2770 | 18 | 1:1000 | Rabbit |  |
| Secondary antibody | **Cat. Number** | **Mr (kDa)** | **Dilution** | **Origin** | **Company** |
| Goat anti-Mouse IgG F(ab´)2 Secondary antibody, HRP | #31436 | - | 1:10 000-1:200 000 | Goat | Thermo Scientific |
| Goat anti-Rabbit IgG F(ab´)2 Secondary antibody, HRP | #31461 | - | 1:10 000-1:200 000 | Goat |  |


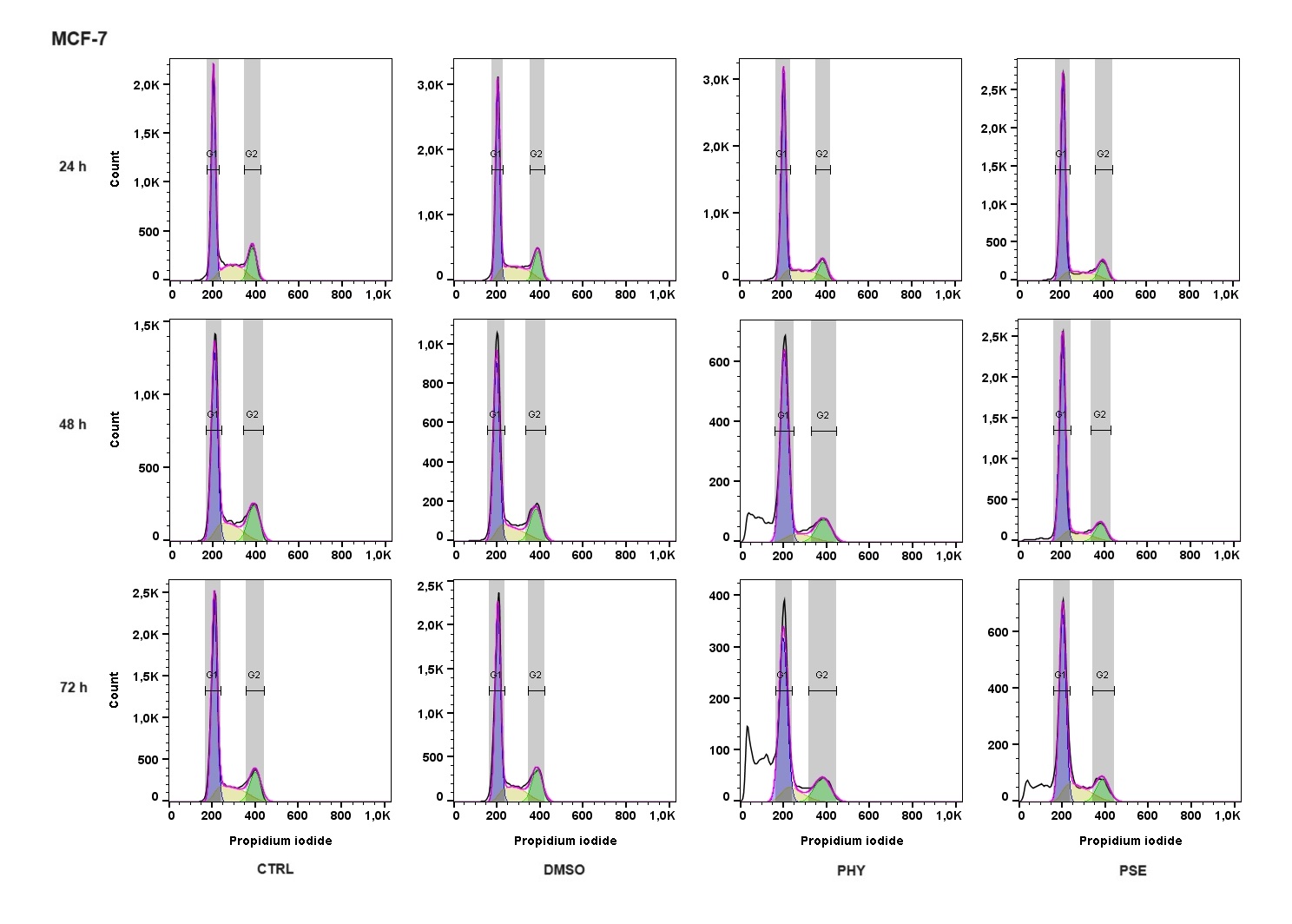


**Figure S1:** Representative diagrams, illustrating cell cycle distribution of MCF-7 cells treated with IC_50_ of PHY and PSE for 24, 48 and 72 hours.


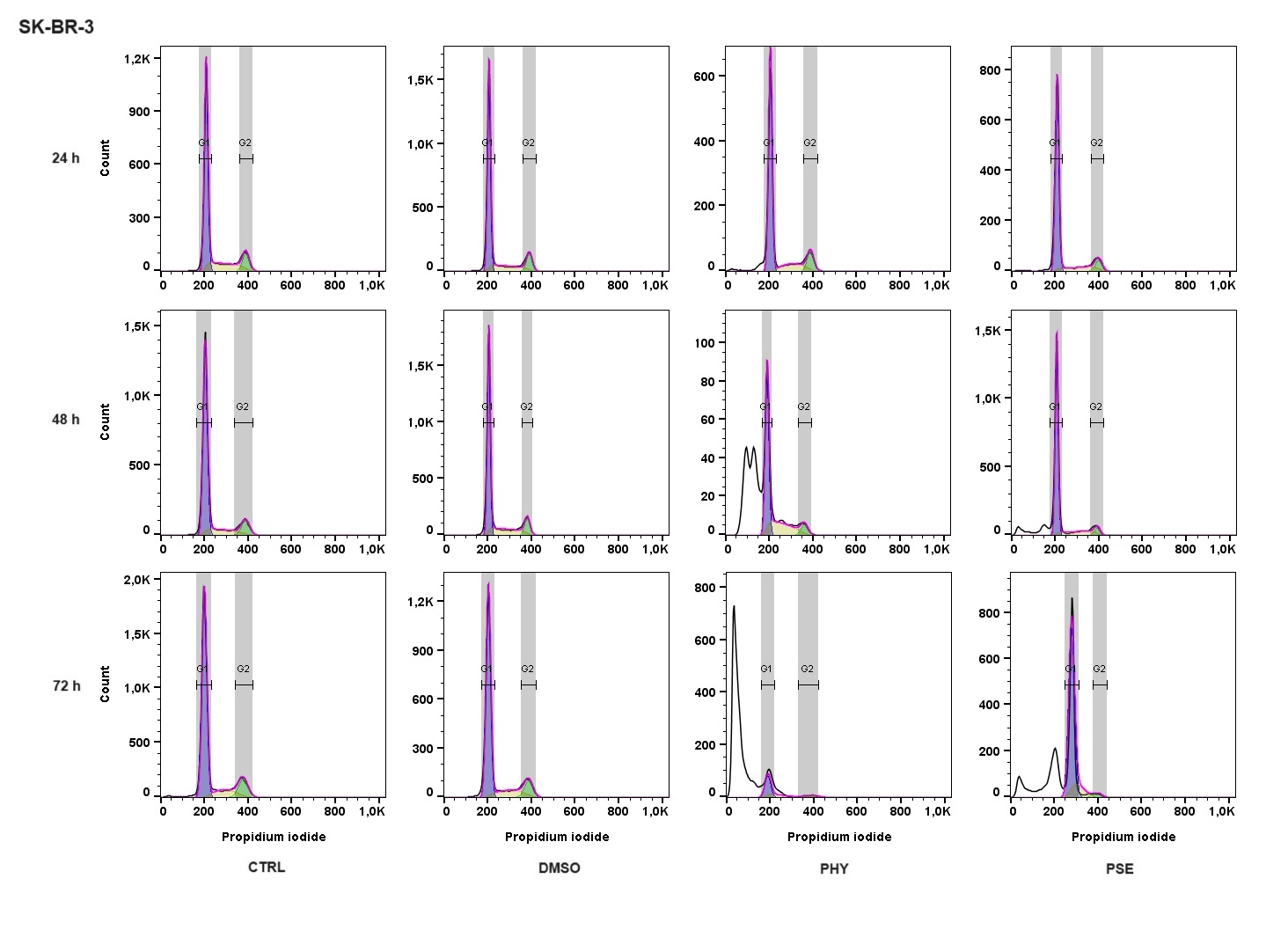


**Figure S2:** Representative diagrams, illustrating cell cycle distribution of SK-BR-3 cells treated with IC_50_ of PHY and PSE for 24, 48 and 72 hours.


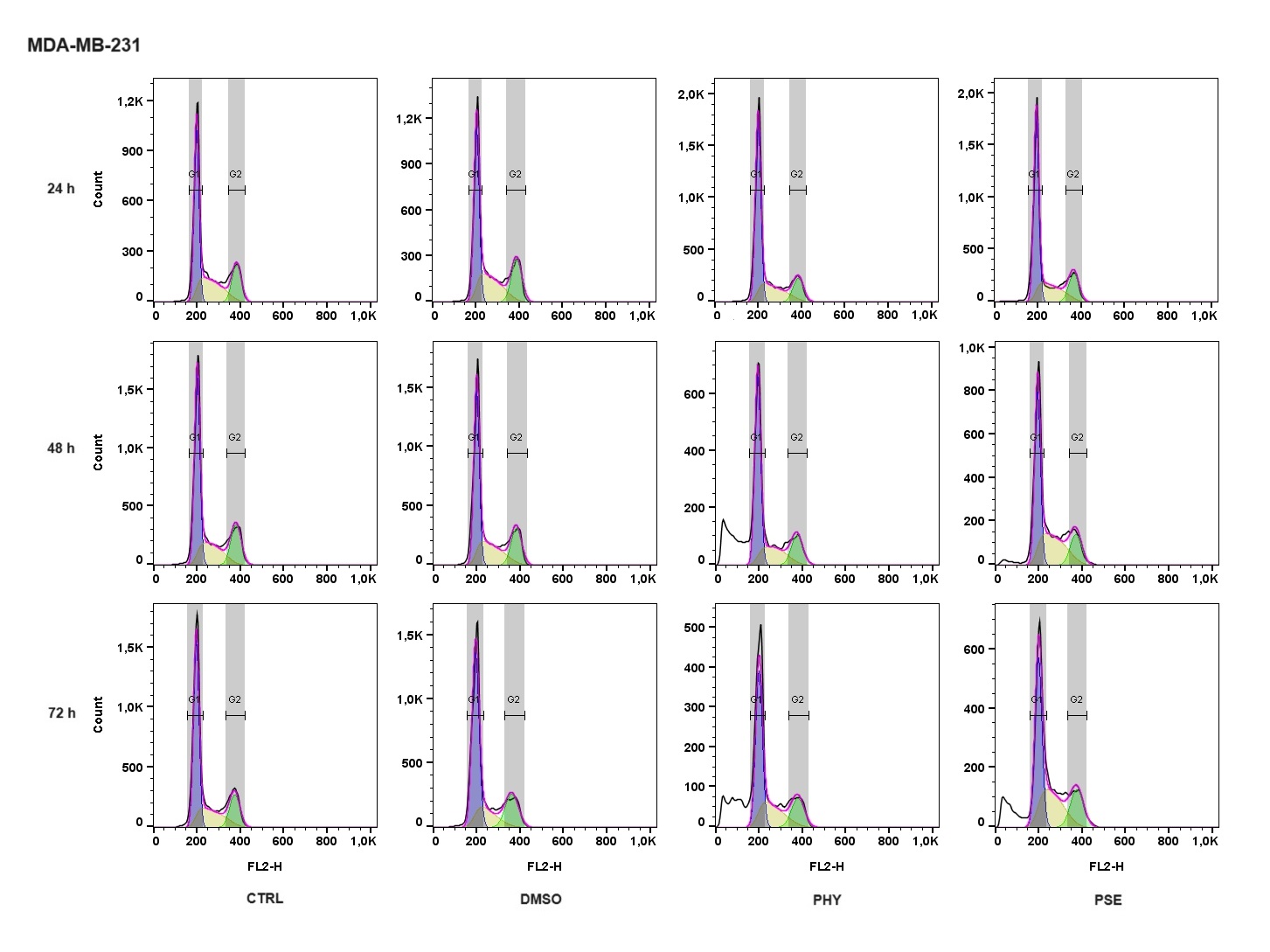


**Figure S3:** Representative diagrams, illustrating cell cycle distribution of MDA-MB-231 cells treated with IC_50_ of PHY and PSE for 24, 48 and 72 hours.


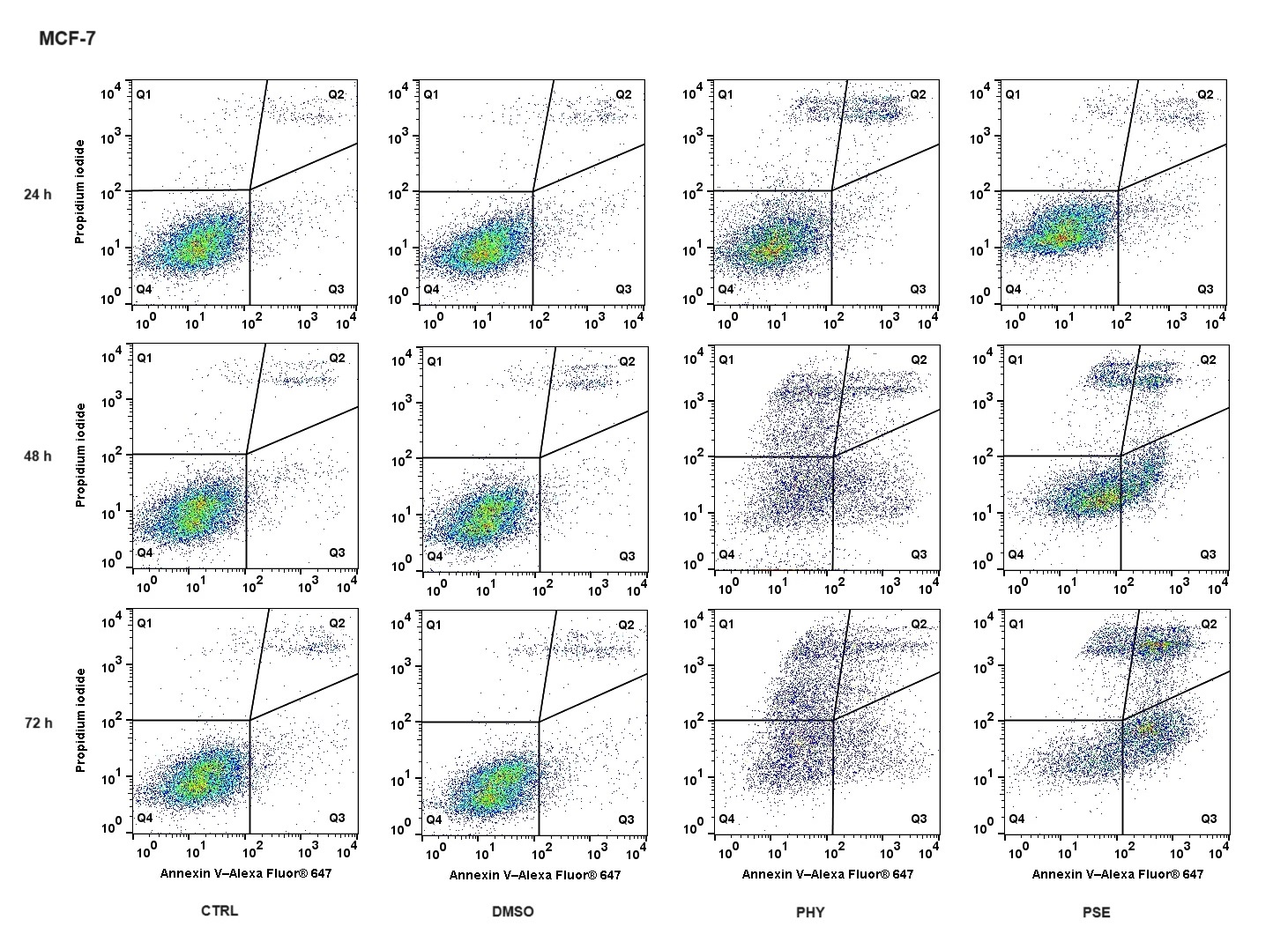


**Figure S4:** Representative dot plots, distribution of cells in live, early apoptotic, late apoptotic and death cell populations in MCF-7 cells incubated with IC_50_ of PHY and PSE for 24, 48 and 72 hours.


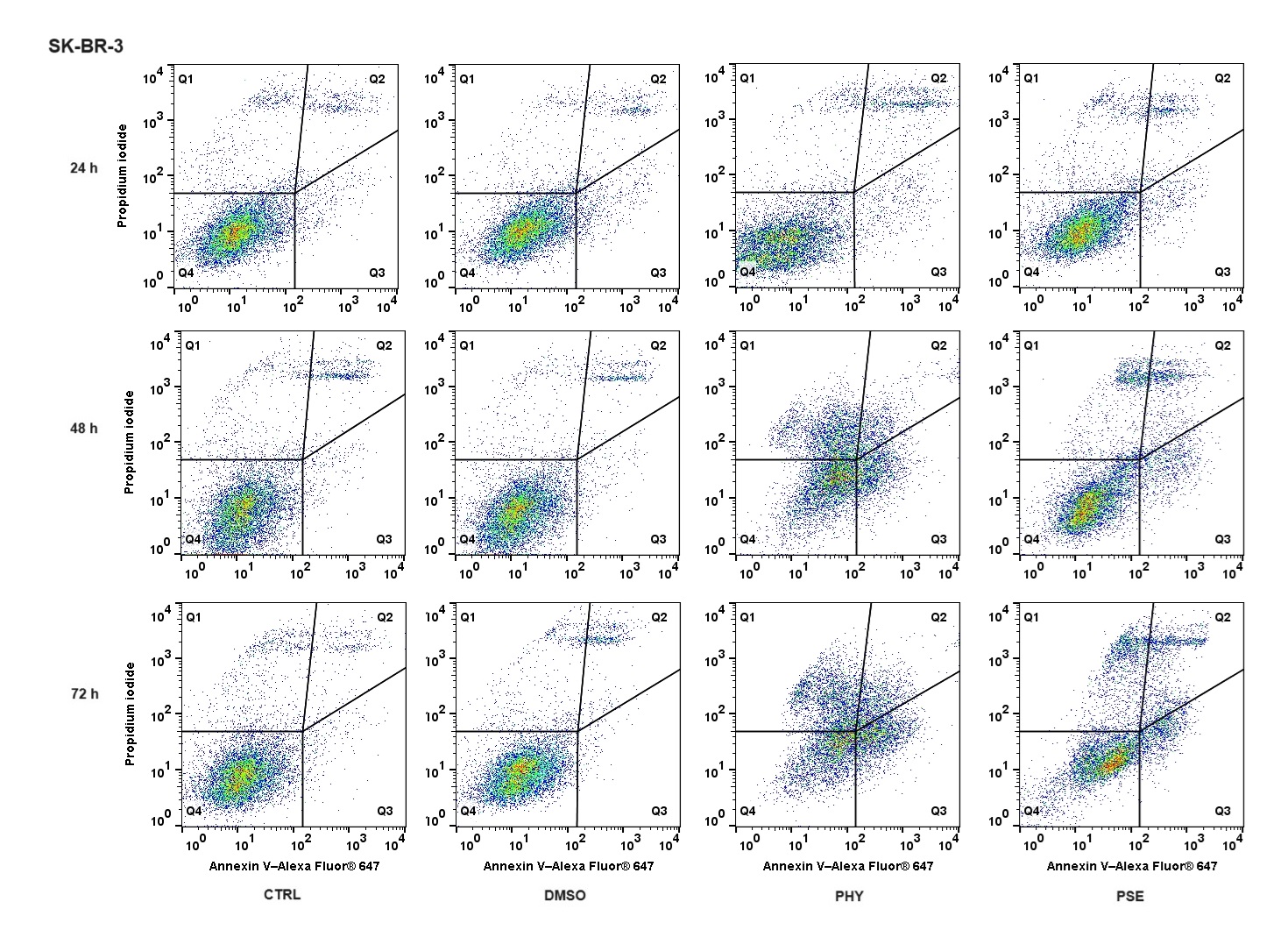


**Figure S5:** Representative dot plots, distribution of cells in live, early apoptotic, late apoptotic and death cell populations in SK-BR-3 cells incubated with IC_50_ of PHY and PSE for 24, 48 and 72 hours.


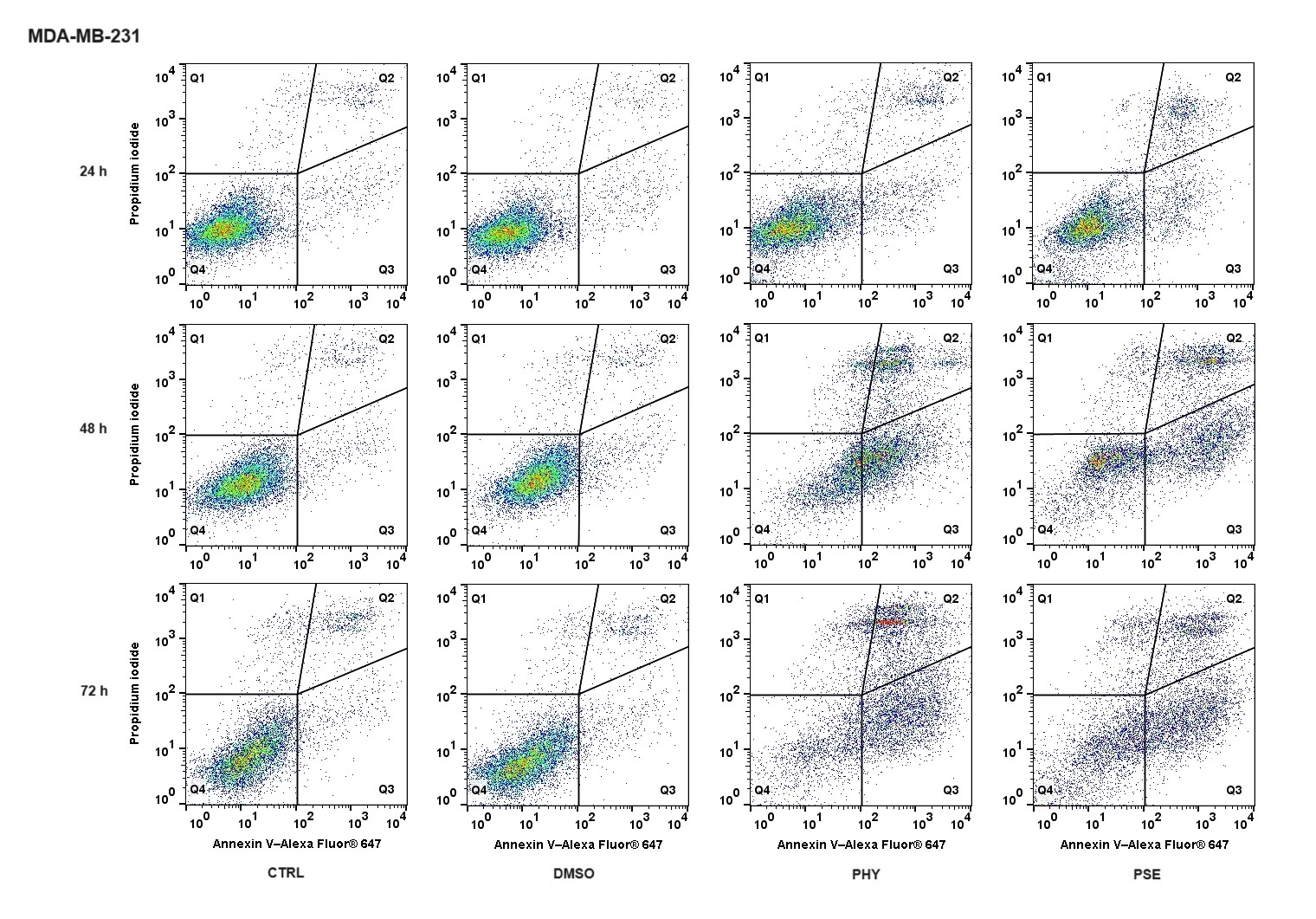


**Figure S6:** Representative dot plots, distribution of cells in live, early apoptotic, late apoptotic and death cell populations in MDA-MB-231 cells incubated with IC_50_ of PHY and PSE for 24, 48 and 72 hours.

**Table S2:** Relative percentage of cells with decreased MMP.

|  |  | **CTRL** | **DMSO** | **PHY** | **PSE** |
| --- | --- | --- | --- | --- | --- |
| **MCF-7** | **24 h** | 6.0 ± 0.60 | 6.8 ± 0.90 | 21.3 ±2.50* | 14.2 ± 1.80* |
|  | **48 h** | 7.1 ± 0.50 * | 11.6 ± 0.80 | 89.1 ± 0.10* | 62.2 ± 2.00* |
|  | **72 h** | 15.1 ± 1.70 | 10.6 ± 1.40 | 93.1 ± 1.50* | 89.3 ± 1.60* |
| **SK-BR-3** | **24 h** | 11.3 ± 0.20 | 14.4 ± 3.30 | 24.6 ± 0.10* | 16.2 ± 0.10 |
|  | **48 h** | 11.9 ± 4.70 | 12.6 ± 4.00 | 96.7 ± 1.20* | 26.0 ± 1.80* |
|  | **72 h** | 15.1 ± 1.50 | 9.8 ± 2.10 | 97.7 ± 0.40* | 73.8 ± 7.30* |
| **MDA-MB-231** | **24 h** | 6.2 ± 0.40 | 5.8 ± 0.20 | 12.6 ± 0.20* | 15.6 ± 0.01* |
|  | **48 h** | 7.6 ± 0.60 | 8.4 ± 0.50 | 86.6 ± 0.10* | 33.5 ± 1.80* |
|  | **72 h** | 9.1 ± 2.40 | 8.2 ± 1.30 | 91.2 ± 2.70* | 62.9 ± 6.80* |

The results are expressed as mean ± standard deviation of three independent experiments. (* p < 0.05 compared to control, based on ordinary one-way ANOVA with Dunnett´s post hoc test).


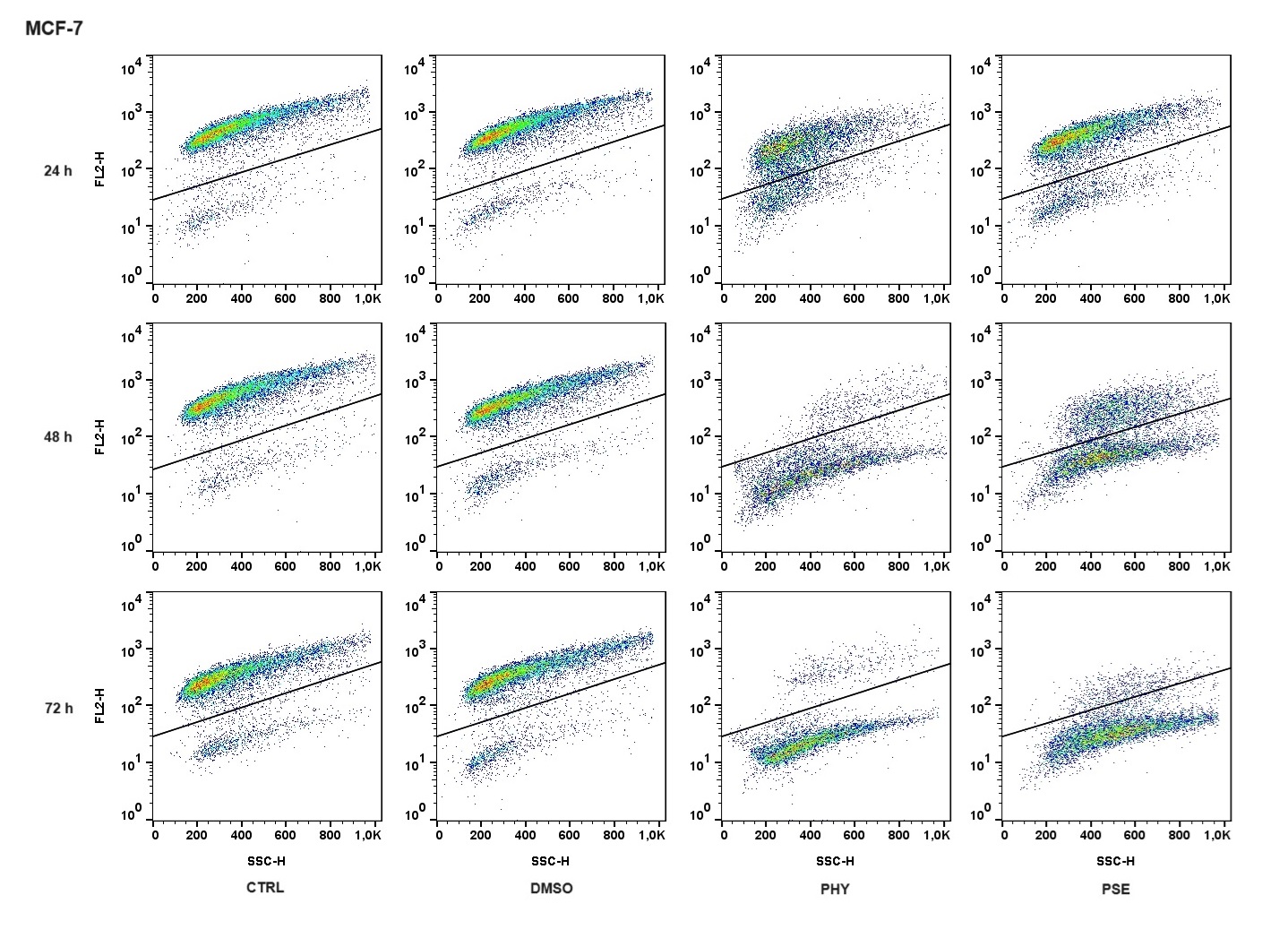


**Figure S7:** Representative dot plots, illustrating changes in mitochondria membrane potential in MCF-7 cells incubated with IC_50_ of PHY and PSE for 24, 48 and 72 hours.


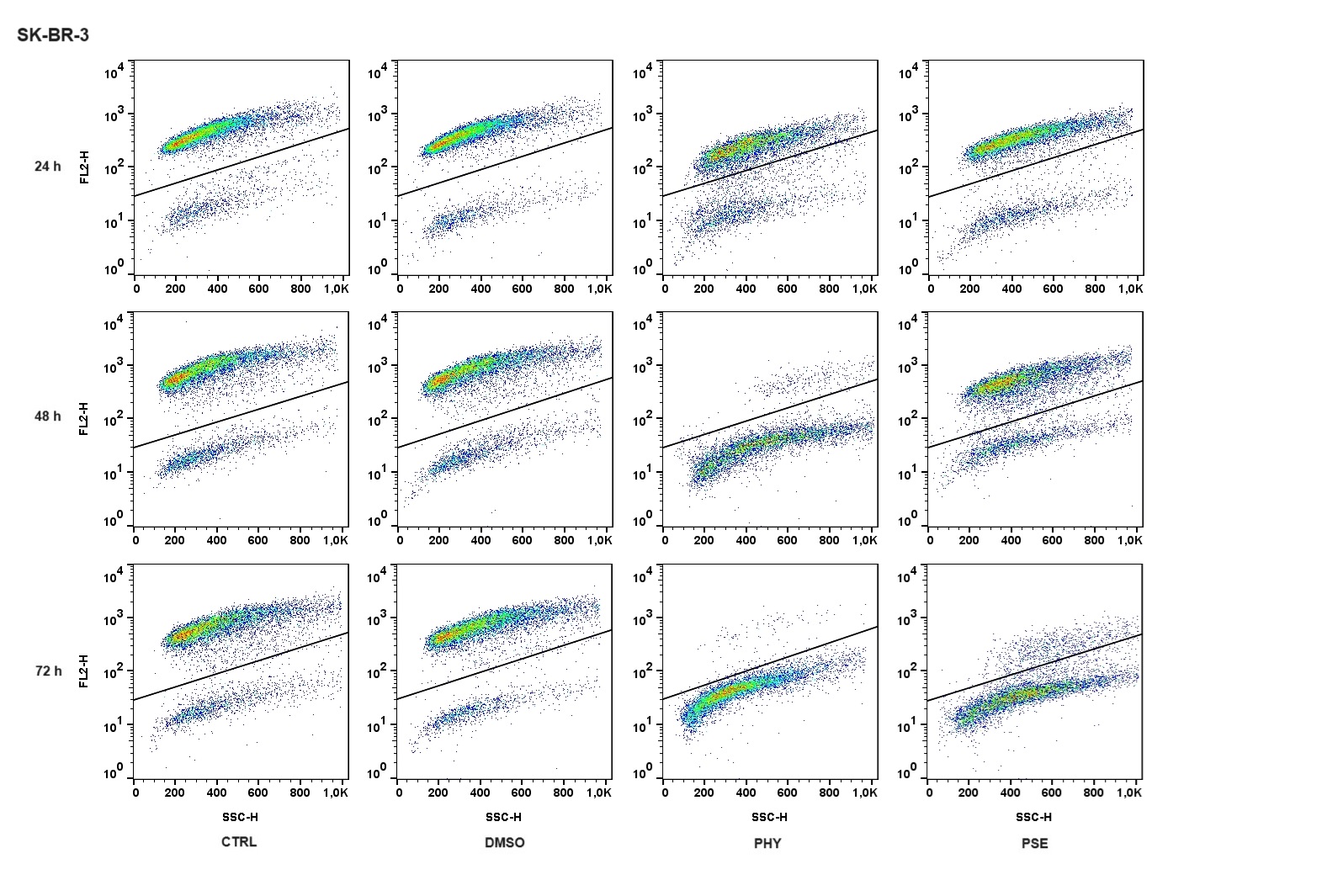


**Figure S8:** Representative dot plots, illustrating changes in mitochondria membrane potential in SK-BR-3 cells incubated with IC_50_ of PHY and PSE for 24, 48 and 72 hours.


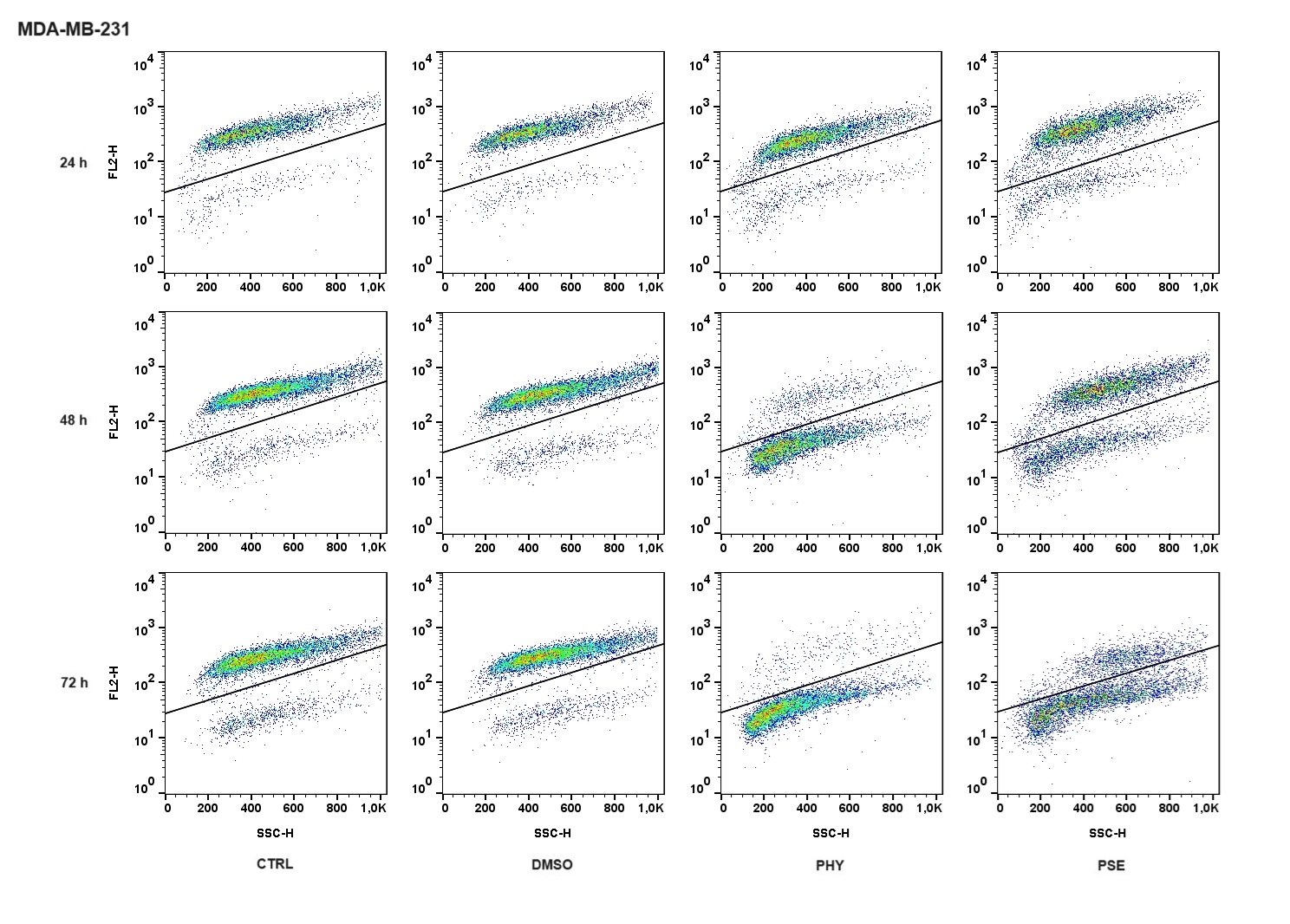


**Figure S9:** Representative dot plots, illustrating changes in mitochondria membrane potential in MDA-MB-231 cells incubated with IC_50_ of PHY and PSE for 24, 48 and 72 hours.

**Table S3:** Relative percentage of cells positive to superoxide. Cells were treated with IC_50_ of PHY/PSE for 3, 6, 24, 48 and 72 h.

|  |  | **CTRL** | **DMSO** | **PHY** | **PSE** | **NAC** | **N+PHY** | **N+PSE** |
| --- | --- | --- | --- | --- | --- | --- | --- | --- |
| **MCF-7** | **3 h** | 3.7 ± 0.8 | 3.9 ± 0.1 | 8.9 ± 2.3* | 7.5 ± 1.2 | 5.1 ± 0.2 | 7.7 ± 2.5 | 5.1 ± 0.5 |
|  | **6 h** | 3.0 ± 0.3 | 2.7 ± 0.3 | 7.0 ± 2.9 | 7.7 ± 1.1 | 4.5 ± 0.9 | 7.7 ± 3.2 | 8.4 ± 1.3* |
|  | **24 h** | 4.1 ± 0.2 | 6.5 ± 0.3 | 25.7 ± 0.5* | 19.9 ± 3.8* | 7.4 ± 0.8 | 17.0 ± 2.8* | 8.6 ± 0.9 |
|  | **48 h** | 4.4 ± 0.1 | 7.2 ± 1.9 | 39.1 ± 2.4* | 51.1 ± 2.4* | 9.6 ± 1.0 | 29.1 ± 4.6* | 33.0 ± 2.5* |
|  | **72 h** | 7.0 ± 0.2 | 4.4 ± 0.7 | 15.1 ± 0.6* | 28.3 ± 2.2* | 8.2 ± 2.2 | 6.0 ± 1.5 | 14.0 ± 3.8* |
| **SK-BR-3** | **3 h** | 2.5 ± 0.1 | 3.9 ± 0.1 | 26.6 ± 3.8* | 15.0 ± 1.1* | 4.5 ± 0.1 | 17.9 ± 3.4* | 7.8 ± 0.7 |
|  | **6 h** | 2.2 ± 0.1 | 2.9 ± 0.5 | 29.6 ± 3.5* | 29.7 ± 4.0* | 3.0 ± 1.1 | 16.3 ± 3.7* | 18.4 ± 1.6* |
|  | **24 h** | 3.8 ± 0.1 | 3.4 ± 0.2 | 31.3 ± 0.8* | 38.5 ± 1.3* | 3.7 ± 1.2 | 19.8 ± 4.1* | 22.8 ± 1.7* |
|  | **48 h** | 2.1 ± 0.2 | 3.2 ± 0.8 | 46.5 ± 0.3* | 37.3 ± 2.7* | 3.5 ± 0.1 | 36.7 ± 0.8* | 16.0 ± 2.3* |
|  | **72 h** | 1.9 ± 0.3 | 3.2 ± 0.7 | 54.3 ± 2.9* | 39.2 ± 4.8* | 5.1 ± 1.9 | 47.5 ± 0.7* | 23.8 ± 0.3* |
| **MDA-MB-231** | **3 h** | 7.1 ± 0.4 | 6.5 ± 0.5 | 6.7 ± 0.3 | 9.4 ± 0.1* | 6.3 ± 1.3 | 7.5 ± 0.1 | 7.7 ± 0.4 |
|  | **6 h** | 4.7 ± 1.1 | 3.6 ± 0.2 | 9.3 ± 8.1* | 14.5 ± 0.6* | 1.9 ± 0.4 | 9.1 ± 1.5* | 8.2 ± 1.0 |
|  | **24 h** | 5.0 ± 0.6 | 3.3 ± 0.2 | 24.8 ± 2.7* | 19.2 ± 0.6* | 5.2 ± 0.2 | 10.8 ± 0.7* | 14.6 ± 0.3* |
|  | **48 h** | 4.4 ± 0.2 | 3.3 ± 0.2 | 30.5 ± 2.3* | 32.7 ± 2.3* | 4.4 ± 0.6 | 10.1 ± 0.2 | 25.0 ± 2.1* |
|  | **72 h** | 3.3 ± 0.2 | 3.9 ± 0.1 | 27.1 ± 0.4* | 44.5 ± 1.0* | 4.6 ± 0.7 | 9.4 ± 2.5* | 13.2 ± 0.5* |

Results are expressed as mean ± standard deviation of independent experiments. (* p < 0.05 compared to DMSO control, based on ordinary one-way ANOVA with Dunnett´s post hoc test).

**Table S4:** Relative percentage of cells positive to nitric oxide. Cells were treated with IC_50_ of PHY/PSE for 3, 6, 24, 48 and 72 h.

|  |  | **CTRL** | **DMSO** | **PHY** | **PSE** | **NAC** | **N+PHY** | **N+PSE** |
| --- | --- | --- | --- | --- | --- | --- | --- | --- |
| **MCF-7** | **3 h** | 1.1 ± 0.1 | 0.5 ± 0.1 | 3.7 ± 1.1* | 3.8 ± 1.2* | 1.6 ± 0.1 | 0.5 ± 0.1 | 0.8 ± 0.3 |
|  | **6 h** | 0.7 ± 0.1 | 2.3 ± 0.3 | 4.6 ± 1.8 | 6.3 ± 0.4* | 0.9 ± 0.1 | 0.5 ± 0.1 | 1.9 ± 0.1 |
|  | **24 h** | 1.3 ± 0.1 | 1.2 ± 0.2 | 11.4 ± 0.4* | 12.4 ± 1.6* | 1.0 ± 0.1 | 2.9 ± 1.4 | 2.9 ± 0.5 |
|  | **48 h** | 0.4 ± 0.1 | 1.5 ± 0.6 | 8.6 ± 2.0* | 10.9 ± 1.9* | 1.0 ± 0.2 | 1.7 ± 0.2 | 3.3 ± 0.1 |
|  | **72 h** | 0.5 ± 0.1 | 2.0 ± 0.7 | 8.7 ± 2.2* | 9.4 ± 1.2* | 1.0 ± 0.1 | 1.6 ± 1.0 | 2.2 ± 0.2 |
| **SK-BR-3** | **3 h** | 1.3 ± 0.4 | 1.2 ± 0.3 | 4.4 ± 1.2* | 3.7 ± 1.2 | 0.5 ± 0.1 | 0.9 ± 0.4 | 1.0 ± 0.7 |
|  | **6 h** | 1.1 ± 0.2 | 1.1 ± 0.5 | 5.9 ± 0.5* | 6.1 ± 1.2* | 1.9 ± 0.2 | 1.0 ± 0.2 | 1.0 ± 0.1 |
|  | **24 h** | 1.1 ± 0.4 | 0.8 ± 0.2 | 4.5 ± 0.9* | 7.1 ± 1.3* | 1.1 ± 0.6 | 1.1 ± 0.6 | 1.0 ± 0.1 |
|  | **48 h** | 0.3 ± 0.1 | 0.9 ± 0.2 | 4.2 ± 0.7* | 8.1 ± 0.6* | 1.6 ± 0.2 | 0.8 ± 0.4 | 2.5 ± 0.8 |
|  | **72 h** | 0.5 ± 0.2 | 1.4 ± 0.9 | 1.0 ± 0.1 | 10.3 ± 0.7* | 1.3 ± 0.4 | 0.5 ± 0.2 | 6.4 ± 0.5* |
| **MDA-MB-231** | **3 h** | 0.6 ± 0.1 | 0.6 ± 0.2 | 0.5 ± 0.1 | 0.6 ± 0.1 | 0.3 ± 0.1* | 0.4 ± 0.1 | 0.3 ± 0.1* |
|  | **6 h** | 0.7 ± 0.1 | 0.6 ± 0.1 | 3.6 ± 0.9* | 0.7 ± 0.1 | 0.9 ± 0.3 | 1.4 ± 0.1 | 0.5 ± 0.3 |
|  | **24 h** | 0.6 ± 0.2 | 0.8 ± 0.2 | 3.5 ± 0.4* | 1.3 ± 0.0 | 0.6 ± 0.1 | 1.0 ± 0.1 | 1.5 ± 0.6 |
|  | **48 h** | 0.6 ± 0.1 | 1.2 ± 0.2 | 5.4 ± 0.6* | 8.9 ± 1.8* | 0.9 ± 0.4 | 0.3 ± 0.1 | 1.3 ± 0.4 |
|  | **72 h** | 0.4 ± 0.1 | 0.5 ± 0.1 | 7.9 ± 0.9 | 27.5 ± 7.6* | 0.6 ± 0.2 | 2.7 ± 0.4 | 12.0 ± 3.5* |

Results are expressed as mean ± standard deviation of independent experiments. (* p < 0.05 compared to DMSO control, based on ordinary one-way ANOVA with Dunnett´s post hoc test).
